# Selective inhibition of BRAF and CRAF sensitizes NF1-deficient malignant peripheral nerve sheath tumors to MEK inhibitors

**DOI:** 10.1186/s10020-025-01353-9

**Published:** 2025-09-29

**Authors:** Jiawan Wang, Arnab Sarkar, Natalia Garcia, Lindy Zhang, Ana Calizo, Alla Lisok, Katia Campos, Funan He, Nishanth Punjaala, Teresa Marple, Kai Pollard, Siyuan Zheng, Calixto-Hope G. Lucas, Vesselina G. Cooke, Christine A. Pratilas, Angelina V. Vaseva

**Affiliations:** 1 Division of Pediatric Oncology, Sidney Kimmel Comprehensive Cancer Center at Johns Hopkins, and Department of Oncology, Johns Hopkins University School of Medicine, Baltimore, MD USA; 2 Greehey Children’s Cancer Research Institute, and Department of Molecular Medicine, The University of Texas Health Science Center, San Antonio, TX USA; 3 Current address: Department of Pediatrics, Oregon Health Science University, SW Pavilion Loop, Portland, OR 3215 USA; 4 Department of Pathology/ Neuropathology, Johns Hopkins University School of Medicine, Baltimore, MD USA; 5 Oncology Drug Discovery, Novartis BioMedical Research, Cambridge, MA USA

**Keywords:** Neurofibromatosis type 1, Malignant peripheral nerve sheath tumor, RAS, RAF, MEK

## Abstract

**Background:**

Treatment for patients with malignant peripheral nerve sheath tumors (MPNST) is an unmet clinical need. Loss of NF1 in MPNST leads to hyperactivation of RAS, however little is known about relevant downstream oncogenic signaling through RAF paralogs and effective targeted therapies in MPNST are still lacking.

**Methods:**

Conditional gene expression, CRISPR-CAS9, and shRNA-mediated knockdown were used to perform gain/loss-of-function experiments to explore the effect of reconstituting the GTPase-activating protein-related domain of NF1 or knockdown of A/B/CRAF kinases on ERK signaling output and MPNST cell growth. Colony formation, cell proliferation and live cells imaging assays were performed to assess cell growth in response to genetic manipulations or drug treatments. Pathway enrichment analysis on RNA sequencing following drug perturbation, efficacy studies in cell-line-derived and patient-derived xenograft models, and immunoblotting/immunohistochemistry were conducted to assess tumor growth and ERK pathway activity in cells or in pharmacodynamic analyses of tumor xenografts.

**Results:**

NF1 loss activates RAS/ERK signaling through B/CRAF, and cell growth and ERK signaling of NF1-MPNST are dependent on B/CRAF, but not ARAF. Genetic or pharmacological inhibition of B/CRAF using a paralog-selective RAF inhibitor (RAFi) significantly potentiates MEK inhibitor (MEKi) treatment through more effective suppression of ERK signaling and proliferation. This is shown in multiple traditional and patient-derived cell line and xenograft models, including those with acquired resistance to MEKi.

**Conclusions:**

These findings contribute preclinical evidence that the combination of paralog-selective B/CRAFi and MEKi is effective in NF1-MPNST and can overcome resistance to single agent MEKi.

**Supplementary Information:**

The online version contains supplementary material available at 10.1186/s10020-025-01353-9.

## Introduction

MPNST arise from the premalignant tumor plexiform neurofibroma (pNF) and are the most common malignancy in patients with neurofibromatosis type 1 (NF1). NF1-MPNST are highly aggressive, with a relatively poor prognosis, and are the leading cause of death in these patients (Rasmussen et al. 2001). Treatment for patients with MPNST remains a challenge due to their relative resistance to chemo- or radiation therapy, challenges associated with complete surgical resection, and propensity for local recurrence and metastasis (Ryan et al. 2012). Survival of patients with NF1-MPNST has not improved for several decades and there is a need for novel therapeutic strategies.

The *NF1* gene encodes the RAS GTPase activating protein (GAP) neurofibromin, a tumor suppressor that negatively regulates RAS activity. Loss of NF1 function leads to hyperactivation of RAS and downstream effector pathways, among which the RAF-MEK-ERK signaling cascade plays a critical role in cell growth and proliferation (Weiss and Shannon 1999). MEKi reduces tumor volume and improves symptoms in the majority of patients with NF1-associated pNF, the precursor to MPNST (Gross et al. 2020). These findings led to the Food and Drug Administration (FDA) approval of the MEKi, selumetinib in 2020 and mirdametinib in 2025 for the treatment of these patients (Casey et al. 2021; Moertel et al. 2024). Similar results have been seen with other agents in this class, namely trametinib and binimetinib (McCowage et al. 2018; Mueller et al. 2020). The ERK signaling pathway has proven the most attractive and relevant therapeutic target for the majority of cancers driven by oncogenic RAS (Ryan et al. 2015; Cox et al. 2014). Unfortunately, single-agent MEKi have limited or short-lived preclinical and clinical activities due to the narrow therapeutic index as well as acquired resistance (Gilmartin et al. 2011; Wang et al. 2021; Wang et al. 2020). These studies suggest a need to combine MEKi with agents targeting the other critical effectors for this malignancy.

The RAF kinases are the RAS effectors responsible for activating the MEK-ERK cascade and function as critical mediators of RAS-driven tumorigenesis (Collisson et al. 2012). Of the three RAF paralogs (A-, B- and CRAF), CRAF has been implicated as the most important for initiating KRAS-driven lung carcinoma (Blasco et al. 2011; Karreth et al. 2011), while it is dispensable for KRAS-driven oncogenesis of pancreatic cancer, indicating that the dependency of tumor growth on RAF family members is context-specific (Eser et al. 2013).

Historically, type 1 RAF inhibitors (RAFi, vemurafenib, dabrafenib and others), which selectively target monomeric BRAF V600 mutants (Joseph et al. 2010), have been ineffective for *BRAF* wild-type and non-V600 mutant cancers due to the paradoxical activation of ERK signaling through induction of RAF dimerization (Lito et al. 2013; Poulikakos et al. 2010). In recent years, however, several type 2 RAFi have been developed to address the mechanistic Limitations of the monomeric binding of type 1 RAFi, through binding and catalytically inactivating both protomers (Noeparast et al. 2018). These inhibitors show promising preclinical anti-tumor activities as single agents in several models of RAS-driven cancers (Peng et al. 2015; Yen et al. 2018; Monaco et al. 2021) and their efficacy is not limited by paradoxical pathway activation. Clinical trials have been initiated with the leading-in-class type 2 RAFi naporafenib (LXH254) (Janku et al. 2024; Braud et al. 2023), and belvarafenib (GDC 5573) (Kim et al. 2019), and preliminary studies recently reported encouraging activity of belvarafenib as a single agent, and of naporafenib plus MEKi trametinib in patients with highly aggressive *NRAS*-mutant melanoma (Braud et al. 2023; Yen et al. 2021). The US FDA recently granted accelerated approval to the brain-penetrant type 2 RAFi tovorafenib as monotherapy for patients with relapsed or refractory *BRAF*-altered pediatric low-grade glioma (Kilburn et al. 2024).

To further improve clinical outcomes with type 2 RAFi, current efforts are focused on the development and testing of combinatorial strategies (NCT03284502, NCT02974725, NCT02607813) evaluating RAF dimer inhibitors in combination with agents targeting MEK or other molecules for the treatment of tumors driven by hyperactive RAS/ERK signaling. Mechanistically, type 2 RAFi plus MEKi sequesters MEK in RAF-MEK complex, uncoupling MEK from ERK (Hong et al. 2021). In support of this, a new pan-RAF/ MEK molecular glue that prevents phosphorylation and activation of MEK by RAF (NST-628), was recently developed and demonstrates activity across a diverse range of cancers driven by ERK signaling alterations (Ryan et al. 2024).

In NF1-deficient MPNST, however, the role of the three RAF paralogs is still unclear, and whether vertical inhibition of ERK signaling via combined inhibition of RAF dimers and MEK will be effective in NF1-associated tumors, is still under investigation. A previous study reported the in vitro potency of the multi-kinase inhibitor sorafenib in cell line models of MPNST which also targets B/CRAF (Ambrosini et al. 2008). We have recently reported that the adaptive response to MEKi in MPNST involves the upregulation of activity of multiple receptor tyrosine kinases (RTK) and the adaptor protein SHP2; and also, have demonstrated that combined inhibition of MEK and MET, or MEK and SHP2 is additive in both in vitro and in vivo models of NF1-MPNST (Wang et al. 2021; Wang et al. 2020). In these studies, upregulation of PDGFRβ and HGF/ MET confers resistance to MEKi in two MEKi-resistant cell line models, respectively, and *RAF1* is genomically amplified specifically in the former resistant model. Further, CRAF protein expression and activity are increased in both MEKi-resistant models (Wang et al. 2021). We therefore hypothesize that the adaptive and acquired resistance to MEKi mediated by upregulation of RTK, at least partially converges on CRAF for sustained activation of ERK signaling, and that pan-RAF inhibition would attenuate the activation of signal transduction from active RAS, as a consequence of the relief of negative feedback induced by MEK inhibition, to MEK.

Here, we demonstrate an inhibitory effect of NF1-GRD (GAP-related domain, residues 1198–1509) fused with RAS-CAAX motif on tumor cell growth and ERK signaling in NF1-MPNST and further prove the growth dependency of NF1-MPNST cells on CRAF and BRAF. A panel of NF1-MPNST cells shows sensitivity to a type 2 paralog selective C/BRAF inhibitor. Restoration of NF1 GAP function or genetic ablation of CRAF or BRAF sensitizes NF1-MPNST cells to the MEKi trametinib. Moreover, combination benefit is observed in in vitro native cell lines as well as in trametinib-resistant models and in vivo cell line and patient-derived tumor xenografts treated with the combination of clinical grade small molecule inhibitors of C/BRAF and MEK. Notably, the in vitro potency of combined type 2 C/BRAFi and MEKi is not obvious in *NF1* wild type (WT) human Schwann cells, the cell lineage to MPNST, suggesting that NF1-deficient MPNST are uniquely sensitive to this combination strategy. Taken together, this study provides evidence for additional therapeutic approaches for treating patients with NF1-deficient tumors.

## Materials and Methods

### In vivo mouse studies

6- to 8-week-old NSG (NOD.Cg-Prkdc^scid^ Il2rg^tm1Wjl^/SzJ, strain #:005557, Jackson Laboratory or Johns Hopkins Oncology Animal Facility) and SCID (C.B-17/IcrHsd-Prkdc^scid^, Envigo) female mice were used for in vivo efficacy studies. All mouse experiments were approved by the Institutional Animal Care and Use Committee (IACUC) at Johns Hopkins (JH) and University of Texas, San Antonio. Minced patient-derived or cell-line derived tumor xenograft fragments from seed mice were implanted subcutaneously close to the sciatic nerves, into study mice (n = 7–8/ arm). Drug treatment was started when tumor size reached roughly 120–200 mm^3^, around five weeks after tumor implantation. Mice were randomized into treatment groups using the randomizr package that arranges animals to achieve the best-case distribution to ensure that each treatment group has a similar mean tumor burden and standard deviation. Vehicle, trametinib (dosed at 0.075 mg/kg and 0.3 mg/kg, QD- once daily), LXH254 (dosed at 25 mg/kg and 50 mg/kg, BID- twice daily), or their combination were administrated by oral gavage, based on mean group body weight, with continuous treatment schedule. The endpoint of the experiment for efficacy studies was considered 4–5 weeks on treatment or the longest tumor diameter of 2 cm as per the approved protocol, whichever occurred first. Mice were monitored daily, and tumors were measured once or twice weekly using calipers in two dimensions, and tumor volume was calculated by: L × W^2^(π/6), where L is the longest diameter and W is the width. Fold change in tumor growth was calculated using the formula: (day X/ day 0)-1. MPNST diagnosis was confirmed by pathologists by reviewing H&E slides from patient-derived primary tumors and mouse xenografts.

### Statistical analysis

Student’s t-test was used to calculate statistical significance. Analyses were considered statistically significant if adjusted *P* < 0.05.

Detailed materials and methods are in the Supplementary Materials and Table S1.

## Results

### NF1 negatively regulates RAS signaling and cell growth in MPNST

**Fig. 1:**
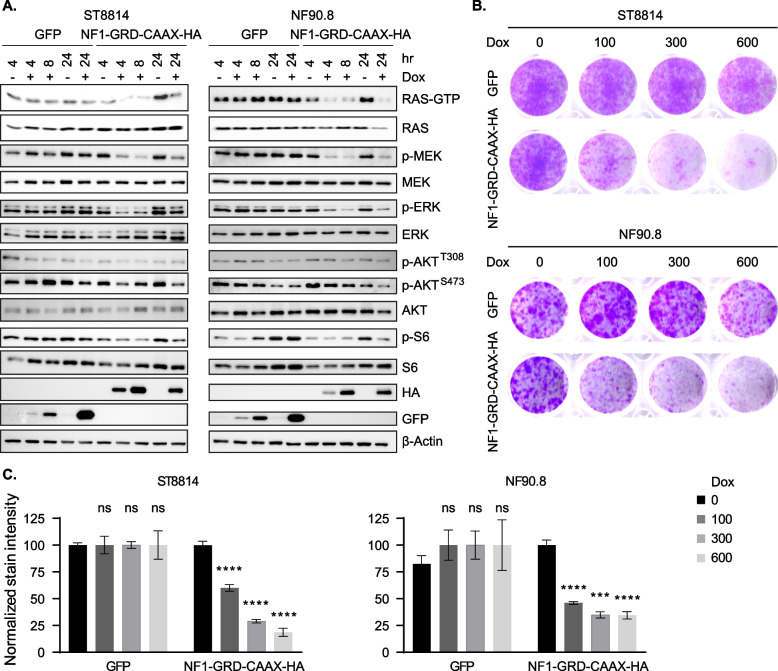
NF1 negatively regulates RAS signaling and cell growth in MPNST. **A** ST8814 and NF90.8 transduced with the lentiviral vectors expressing doxycycline-inducible GFP or HA-tagged NF1-GRD fused with RAS CAAX motif. Cells were treated with vehicle or 300 ng/ml doxycycline (Dox) for 4, 8 and 24 h and signaling intermediates involved in RAS effector pathways (ERK and AKT/ mTOR) were detected using immunoblot. Representative blots from three biological replicates are shown. **B** The cells as described in **A** were treated with Dox at 0, 100, 300 and 600 ng/ml, and the clonogenic assay was performed after 10–15 days by washing cells with PBS, fixing with 10% neutral buffered formalin (NBF) and then staining with 0.1% crystal violet. Representative images from three biological replicates are shown. **C** Bar graphs summarizing crystal violet stain intensity in ST8814 and NF90.8 cells expressing Dox-inducible GFP or NF1-GRD-CAAX normalized to non-treated control. Graphs correspond to Fig. 1B. Error bars represent SEM from three biological replicates. Each treatment group was compared to non-treated control and statistical difference was determined using unpaired t-test with GraphPad Prism software. ***p < 0.001; **** *p* < 0.0001

It is well established that NF1 inactivation upregulates RAS/ERK signaling (Nissan et al. 2014; Maertens et al. 2013). *NF1* is genetically altered in ~ 90% of MPNST (Brohl et al. 2017). Accordingly, RAS hyperactivation is critical to the pathogenesis of *NF1*-mutant tumors (Malone et al. 2014). Despite the existence of NF1 RAS-dependent and independent functions, GRD alone is sufficient to diminish RAS activity (Ballester et al. 1990) and inhibit cell hyperproliferation in NF1 null mouse models (Ismat et al. 2006). The GAP activity of NF1 isoform 2 (NP_000258.1, lacking exon 23a) is tenfold higher than isoform 1 (Nguyen et al. 2017). To prove this concept, we cloned the GRD of NF1 isoform 2 and reconstituted NF1 GAP function by ectopically expressing NF1-GRD fused with HRAS CAAX motif that is required for membrane targeting (Bai et al. 2019), in two NF1-null MPNST cell lines ST8814 and NF90.8 (Figs. 1 and S1). HA-tagged NF1-GRD-CAAX reduced RAS-GTP levels and led to significant inhibition of p-MEK and p-ERK in a time (Figs. 1A and S1A) and dose (Figure S1B and S1C) dependent manner. While inhibition of p-AKT and p-S6 was less prominent, indicating that MEK-ERK signaling is likely the major effector of active RAS in these cells. As a consequence of reduced RAS effector signaling, re-expression of NF1-GRD-CAAX also inhibited cell growth in the two cell lines (Figs. 1B and C). With this in mind, we also knocked out *NF1* in the sporadic MPNST cell line STS26T harboring *NF1* WT/ *BRAF* V600E by using sg-RNA guided CRISPR-Cas9 technique. We observed an increase in the activity of CRAF, as evidenced by elevation in p-CRAF Ser338, as well as other RAS effectors, in particular p-MEK and p-ERK, and to a lesser extent p-AKT and p-S6, upon *NF1* depletion (Figure S1D). Although the control cells in this experiment were parental cells not infected with non-targeting sgRNA vector, the data were consistent with NF1-GRD-CAAX expression experiments above. These findings recapitulate the observations in *BRAF*-mutant melanoma (Maertens et al. 2013), and suggest the importance of CRAF and MEK-ERK signaling in NF1 null cells. Overall, the results indicate the driver role of impaired regulation of RAS due to loss of NF1 function in MPNST, and that NF1 GRD alone is able to perform GAP function to decrease RAS activity and downstream effector signaling, particularly RAF-MEK-ERK.

### NF1-MPNST cells are sensitive to C/BRAF genetic depletion or pharmacological inhibition

All three RAF paralogs share a conserved domain architecture and perform a central role as MAP3K in RAS/ERK signaling (Terrell and Morrison 2019), but they exhibit differences in basal kinase activity and may perform functional redundancy and compensation when other paralogs are inhibited (Matallanas et al. 2011). CRAF is a crucial mediator of resistance to RAF/MEK inhibition in *BRAF*-mutant melanoma and colorectal cancer models (Montagut et al. 2008; Whittaker et al. 2015), through sustained ERK pathway activation. NF1 knockdown in *BRAF*-mutant melanoma cells leads to concomitant increased activity of RAS and CRAF, and combined knockdown of NF1 and CRAF restores the sensitivity of resistant cells mediated by loss of NF1 to BRAFi (Whittaker et al. 2013), suggesting the pivotal role of CRAF in ERK signaling transduction driven by loss of NF1.

**Fig. 2:**
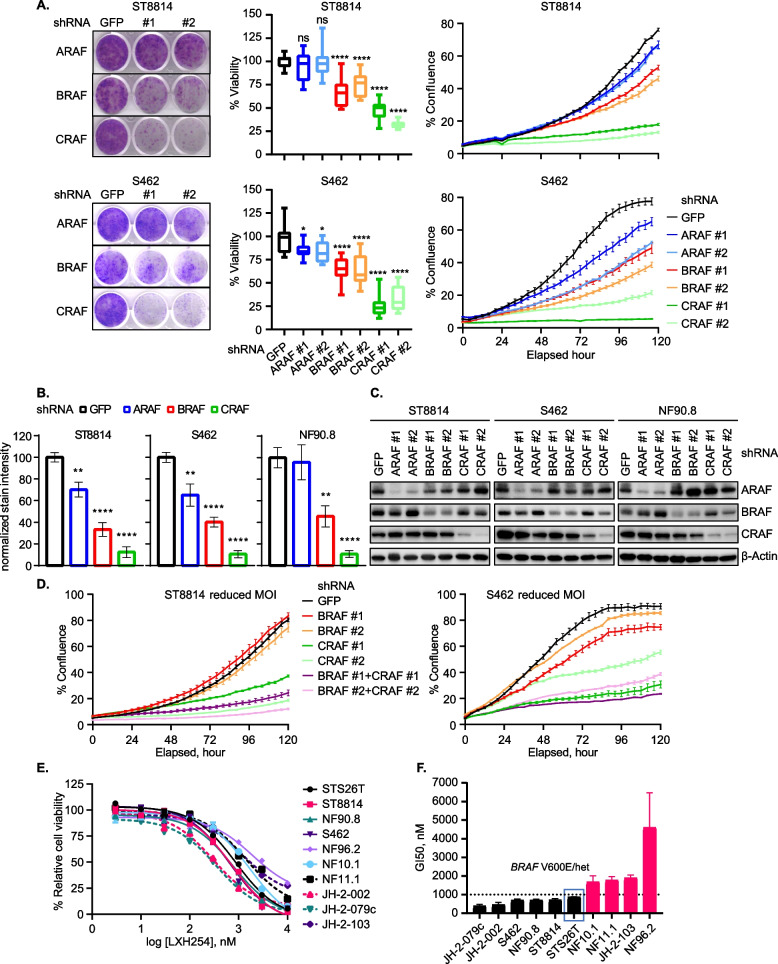
NF1-MPNST cells are sensitive to C/BRAF genetic depletion or pharmacological inhibition. **A** Left: Cells transduced with lentiviral vectors expressing shRNA targeting *GFP* or two independent sequences targeting *ARAF*, *BRAF* or *CRAF* were subjected to crystal violet staining. Cells were plated at low density four days after virus transduction and after approximately 10 days, cell growth was visualized with crystal violet staining. Representative images from three biological replicates are shown; Middle: Cell viability assay normalized to *GFP* shRNA-expressing cells using MTT assay. Cells were seeded in 96 well plates 4 days after virus transduction, and viability was assessed after 96 h. Error bars represent SEM from three biological replicates with three technical replicates in each setup. Each experimental group was compared to sh*GFP* group and statistical difference was determined using unpaired Student t-test with GraphPad Prism software. ns = not significant, * *p* < 0.05, ** *p* < 0.01, *** *p* < 0.001, **** *p* < 0.0001; Right: IncuCyte phase contrast imaging of cells treated as in middle panel. Shown are representative experiments from three biological replicates with three technical replicates in each setup. **B** Bar graphs summarizing crystal violet stain intensity of *ARAF*, *BRAF*, or *CRAF* shRNA-expressing NF1-MPNST cells normalized to sh*GFP* control. Graphs correspond to Figs. 2A left and S2A left. Error bars represent SEM from three biological replicates with three technical replicates in each setup. Each experimental group was compared to sh*GFP* control and statistical difference was determined using unpaired t-test with GraphPad Prism software. **p < 0.01, **** p < 0.0001. **C** Western blots for A/B/CRAF proteins in NF1-MPNST cells expressing *GFP* or *A/B/CRAF* shRNAs. Indicated cells were treated as in Fig. 2A and 4 days after virus transduction, levels of RAF knockdown were assessed using western blotting. Representative blots from three biological replicates are shown. **D** Cell confluency was monitored using IncuCyte phase contrast imaging in ST8814 and S462 cells transduced with lentiviral vectors expressing sh*GFP*, sh*BRAF* #1 and #2, sh*CRAF* #1 and #2, sh*BRAF* #1 + sh*CRAF* #1 and sh*BRAF* #2 + sh*CRAF* #2. Cells were seeded in 96 well plates 4 days after virus transduction. % confluency was calculated using the Incucyte Live-Cell Analysis System and graphed as a function of elapsed time. A reduced multiplicity of infection (MOI) was used to detect the difference of % confluency between sh*CRAF* and sh*BRAF* + sh*CRAF infected cells*. Shown are representative experiments from three biological replicates with three technical replicates in each setup. **E** One sporadic STS26T and nine NF1-associated MPNST cell lines were treated with DMSO or increasing doses of LXH254 for five days, and cell viability was assessed using CCK-8 assay. The % relative cell viability dose response curves were plotted as a function of log_10_ transformed LXH254 concentrations after background subtraction and normalized to DMSO control. **F** Growth inhibition of 50% (GI50) was calculated using GraphPad prism 10. The sporadic *NF1* WT MPNST cell line STS26T has a mutation in *BRAF* V600E/het as shown in the bar graph and serves as a reference for sensitivity to the RAFi LXH254. The dash line at 1000 nM was set to group the ten cell lines into two clusters, six lines (black bars) as very sensitive and four lines (pink bars) as partially sensitive to LXH254 single agent. Error bars represent SEM from two biological replicates with three technical replicates in each setup

To understand the role of the three RAFs in NF1-deficient MPNST cells, we introduced lentivirus-based shRNA-mediated knockdown of A/ B/ CRAF using two independent shRNAs validated previously (see Supplementary Methods). When compared to the sh*GFP* control, CRAF knockdown most potently inhibited cell growth, while BRAF knockdown had a moderate effect, and ARAF knockdown had only a subtle impact on cell growth (Figs. 2A-B, S2A-F). A/B/CRAF protein levels were reduced by shRNA expression (Figs. 2C and S2G). To further compare the effects of sh*CRAF* alone versus sh*CRAF* plus sh*BRAF* on cell growth and ERK signaling, we reduced the multiplicity of infection in transduction to achieve less potent knockdown of BRAF and CRAF in ST8814 and S462 cells. The less potent knockdown of BRAF or CRAF resulted in almost complete lack of growth inhibition by sh*BRAF* in both cell lines, and less acute but still significant growth inhibition by sh*CRAF*, *w*hile combined knockdown of BRAF and CRAF elicited better potency than CRAF knockdown alone (Figs. 2D, S2H-K). We did not perform triple knockdown of the three RAF kinases given that previous data have shown increased toxicity in mice with elimination of all three RAF paralogs (Sanclement et al. 2018).

We therefore assessed the in vitro efficacy of a potent and selective inhibitor of BRAF and CRAF― LXH254 (Monaco et al. 2021) on a panel of ten MPNST cell lines, including both widely available traditional cell lines as well as JH patient-derived lines that represent the genomic diversity of MPNST (Fig. 2E). These ten cell lines exhibited sensitivity to LXH254 single agent with GI50 values less than 5 µM. As compared to a sporadic line STS26T with *NF1* WT/ *BRAF* V600E as a sensitive control, five of the nine NF1-deficient cell lines had GI50 < 1 µM while four other lines had GI50 between 1 µM and 5 µM (Fig. 2F). A previous study demonstrated that loss of ARAF expression via functional deletion sensitizes RAS-mutant cells to LXH254 (Monaco et al. 2021). To test whether RAF protein expression levels were associated with LXH254 sensitivity in NF1-mutant MPNST cells, we detected the expression levels of ARAF, BRAF and CRAF on this panel of cell lines (Figure S2L-M). An analysis of steady-state ARAF, BRAF or CRAF levels (normalized to GAPDH) with GI50 of LXH254 did not reveal a correlation between RAF paralog expression and LXH254 GI50 in MPNST (Figure S2N). These results confirmed the critical role of CRAF in NF1 biology and demonstrated that either genetic depletion of CRAF/BRAF (but not ARAF) or pharmacological inhibition using type 2 RAFi impaired the growth of NF1-MPNST cells, underscoring the role of CRAF/ BRAF as potential targets for NF1-MPNST.

### Re-expression of NF1 GRD or CRAF/ BRAF genetic depletion sensitizes NF1-MPNST cells to MEK inhibition

**Fig. 3:**
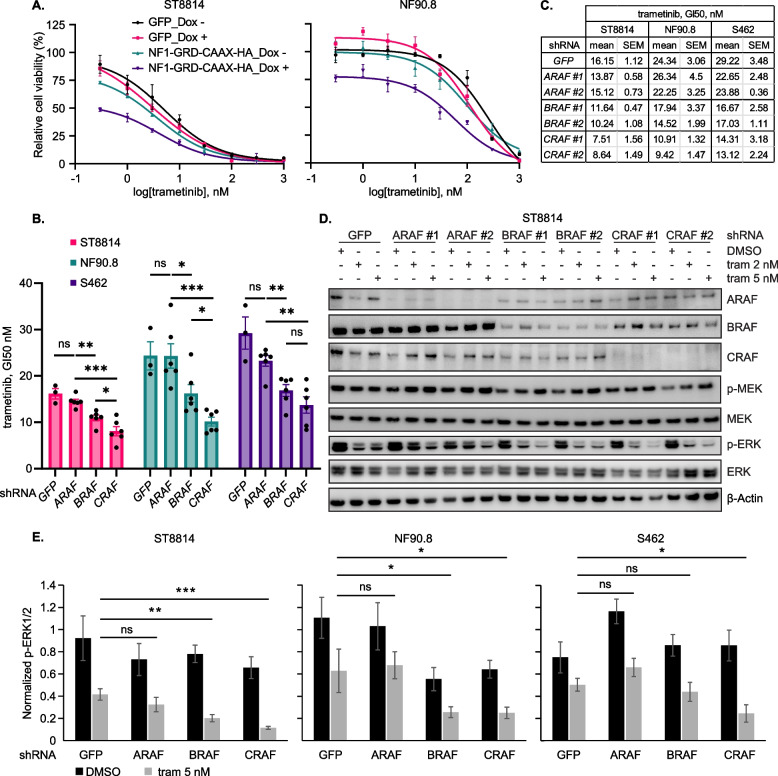
Re-expression of NF1 GRD or CRAF/ BRAF genetic depletion sensitizes NF1-MPNST cells to MEK inhibition. **A** ST8814 and NF90.8 cells as described in Fig. 1A. were treated with DMSO and increasing doses of trametinib for five days and then cell viability was assessed using CCK-8 assay. Shown are representative graphs from three biological replicates with three technical replicates in each setup. **B** Three cell lines as described in Fig. 2A. and S2A. were treated with DMSO and increasing doses of trametinib for five days and then cell viability was assessed. To detect sensitization, viability was normalized to corresponding DMSO control for each shRNA group and GI50 values of trametinib were calculated with GraphPad Prism 10 software. Error bars represent SEM from three biological replicates with three technical replicates in each setup. sh*A/B/CRAF* experimental groups were compared to sh*GFP* groups or between each other using unpaired t-test with GraphPad Prism software. *p < 0.05, **p < 0.01, ***p < 0.001, **** *p* < 0.0001, ns = not significant. **C.** Table summarizing GI50 values from the bar graphs in B. **D** ST8814 cells as described in Fig. 2A. were treated with DMSO or trametinib (tram, 2 and 5 nM) for 24 h. Proteins involved in ERK signaling were detected using immunoblot. Shown are representative images from three biological replicates. **E** Bar graphs summarizing p-ERK protein levels normalized to actin levels in NF1-MPNST cells following A/B/CRAF knock down and treatment with DMSO or 5 nM trametinib. Graphs correspond to Figs. 3D and S3D. Protein levels were quantified from blots using ImageJ. Error bars represent SEM from three (ST8814 and NF90.8) or five (S462) biological replicates. 5 nM trametinib treatment of each RAF knockdown group was compared to 5 nM trametinib treatment of sh*GFP* control and statistical difference was determined using unpaired t-test with GraphPad Prism software. *p < 0.05, **p < 0.01, ***p < 0.001, ns = not significant

MEKi are effective in treating people with NF1-associated clinical manifestations, including pNF (Gross et al. 2020) and low-grade gliomas (Fangusaro et al. 2019), but have demonstrated limited activity in preclinical models of NF1-MPNST (Wang et al. 2021; Wang et al. 2020). To ask whether restoration of NF1 GAP function enhances sensitivity to MEKi, we treated ST8814 and NF90.8 cells transduced with the lentiviral vectors encoding GFP or NF1-GRD, with increasing doses of trametinib for five days. Ectopic expression of NF1 GAP reduced baseline cell growth and potentiated the effect of trametinib in terms of cell proliferation inhibition (Figs. 3A and S3A-B). Given that there are no agents directly targeting loss of NF1 GAP and the critical role of CRAF/BRAF in ERK signaling relay driven by loss of NF1, we next tested the impact of genetic ablation of the three RAF paralogs on MEKi sensitivity. In the three NF1-MPNST cell lines tested, sh*CRAF* decreased trametinib GI50 by 54% on average, sh*BRAF* by 36% and sh*ARAF* by 10%, compared to sh*GFP* (Figs. 3B-C and S3C), through more potent inhibition of ERK signaling when CRAF or BRAF (but not ARAF) knockdown was combined with MEKi (Figs. 3D-E and S3D). In line with previous studies (Imoto et al. 2023), we also found that targeting one RAF can affect the expression of other RAF proteins . In all the three NF1-MPNST cell lines, BRAF expression was significantly downregulated by CRAF knockdown, relative to sh*GFP* controls and ARAF knockdown cells. Therefore, the enhanced effects of CRAF knockdown may stem from the downregulation of both BRAF and CRAF (Figs. 3D-E, and S3D). These data suggested that co-targeting of CRAF/BRAF and MEK using clinically available type 2 RAFi and MEKi may provide further synergy in NF1-MPNST.

### The combined inhibition of RAF and MEK is active in vitro and elicits more potent suppression of ERK signaling

Both adaptive (short-term treatment) and acquired (long term exposure) resistance to MEKi involves reactivation of ERK signaling by upregulation of RTK (Wang et al. 2021). MEKi suppress ERK signaling through reducing ERK phosphorylation, but this results in loss of negative feedback, and MEKi adaptively induce RAS activity (RAS-GTP), BRAF-CRAF heterodimerization and p-MEK to reactivate ERK signaling, accounting for short-lived response to MEKi in RAS-mutant and NF1 loss tumors (Nissan et al. 2014; Lamba et al. 2014). Furthermore, CRAF suppression is synthetic lethal with MEK inhibition in KRAS-mutant colorectal as well as in lung cancer cells, and prolonged suppression of ERK signaling is achieved by dual blockade of RAF and MEK (Lamba et al. 2014).

**Fig. 4:**
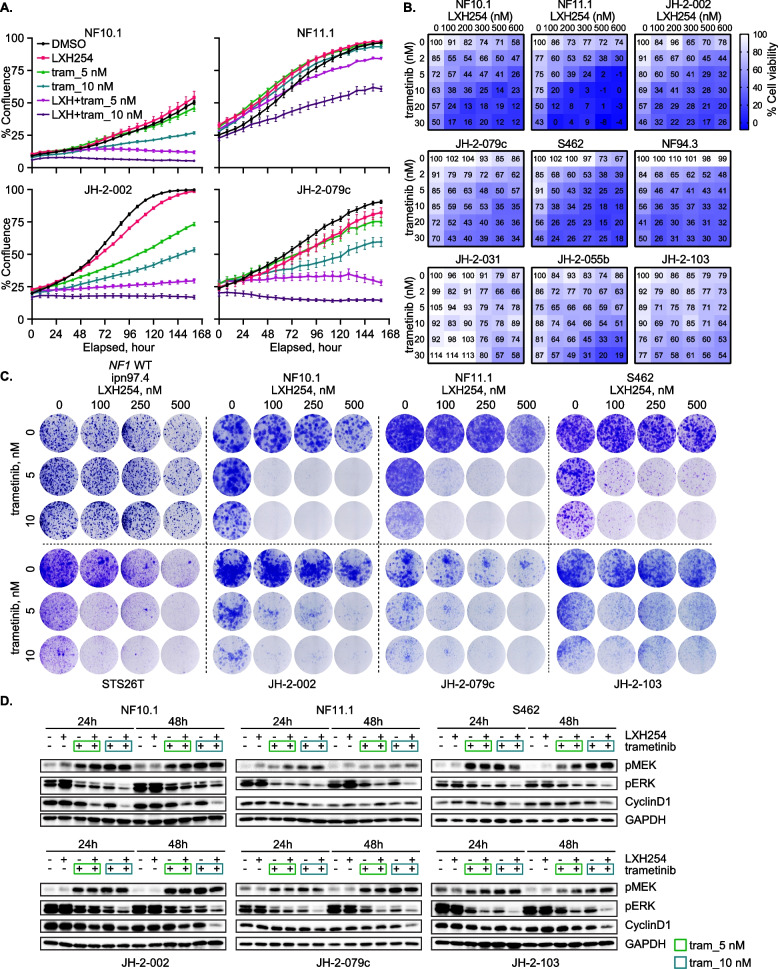
The combined inhibition of RAF and MEK is active in vitro and elicits more potent suppression of ERK signaling. **A** % confluency of four NF1-MPNST cell lines treated with DMSO, 250 nM LXH254, 5 and 10 nM trametinib or their combination, was monitored using IncuCyte as described for Fig. 2A. Cells were plated in 96-well plates, the next day treated, and imaging started. Shown are representative experiments from three biological replicates with three technical replicates in each setup. **B** Nine NF1-MPNST cell lines were treated with DMSO, increasing concentrations of LXH254 and/or trametinib for 5 days, and cell viability was determined using MTT assay. Shown are representative experiments from three biological replicates with three technical replicates in each setup. **C** One *NF1* WT (ipn97.4), one sporadic (STS26T) and six NF1-MPNST cell lines were treated with DMSO, increasing concentrations of LXH254 and/or trametinib for about two weeks. Cell growth was assessed by crystal violet staining. Shown are representative images from two to four biological replicates for each cell line. **D** Six NF1-MPNST cell lines were treated as described in **A** for 24 and 48 h and the indicated proteins were detected using immunoblot. Shown are representative images from two to three biological replicates for each cell line

As shown in Figs. 1–3, the function of NF1 GAP and CRAF/BRAF is pivotal in ERK signaling in MPNST. With this in mind, we evaluated the in vitro potency of the combination of LXH254 and trametinib in models of NF1-MPNST. Combination benefit with regard to cell growth/ proliferation inhibition was achieved with LXH254 plus trametinib in NF1-null MPNST cell lines using three complementary assays, IncuCyte real-time monitoring of cell confluence (Figs. 4A, S4A-B), cell viability assay (Figs. 4B and S4C) and 2-dimensional colony formation assay (Figs. 4C and S4D). However, this combination elicited minimal effects on *NF1* WT human Schwann cells (ipn02.3-2λ and ipn97.4) (Figs. 4C and S4E-F), the cell lineage thought to give rise to MPNST (Li et al. 2016). p-ERK and Cyclin D1 were additively inhibited by the combination, and we also observed an increase in p-MEK driven mainly by trametinib as a consequence of signaling adaptation (Figs. 4D and S4G). In addition, trametinib reduced the level of p-CRAF Ser289/296/301 (associated with CRAF inactivation) at 24- and 48-h treatment, suggesting activation of CRAF after MEKi due to relief of negative feedback (Figure S4H). Since studies have indicated that combinations of different classes of RAF inhibitors could be synergistic in both RAF WT and *BRAF* V600E cancer cells (Rukhlenko et al. 2021), we also compared the efficacy of combining type 2 RAFi with type 1 RAFi (dabrafenib and vemurafenib) or with MEKi (Figure S4I-K). The combination of type 2 RAFi plus MEKi demonstrated the greatest potency, further supporting the investigation of type 2 RAFi plus MEKi in NF1-MPNST.

### The combined inhibition of RAF and MEK demonstrates synergy in MEKi-resistant models

We previously established two models (ST8814Res and NF90.8Res) with acquired resistance to trametinib, in which mechanisms of acquired resistance are mediated by HGF/MET and PDGFRβ, respectively (Wang et al. 2021). Through targeted gene sequencing, we identified *RAF1* genomic amplification, particularly in NF90.8 resistant cells, relative to the parental control (Wang et al. 2021). Trametinib treatment notably induced CRAF total protein expression and activity as measured by p-CRAF Ser338 in both parental lines but had subtle effects on resistant lines. The level of CRAF protein is markedly increased in NF90.8 resistant versus parental cells, regardless of trametinib addition, in line with genomic amplification of *RAF1* in this resistant model (Wang et al. 2021), consolidating the role of CRAF in mediating adaptive (short term) and acquired (long term) resistance to trametinib.

**Fig. 5:**
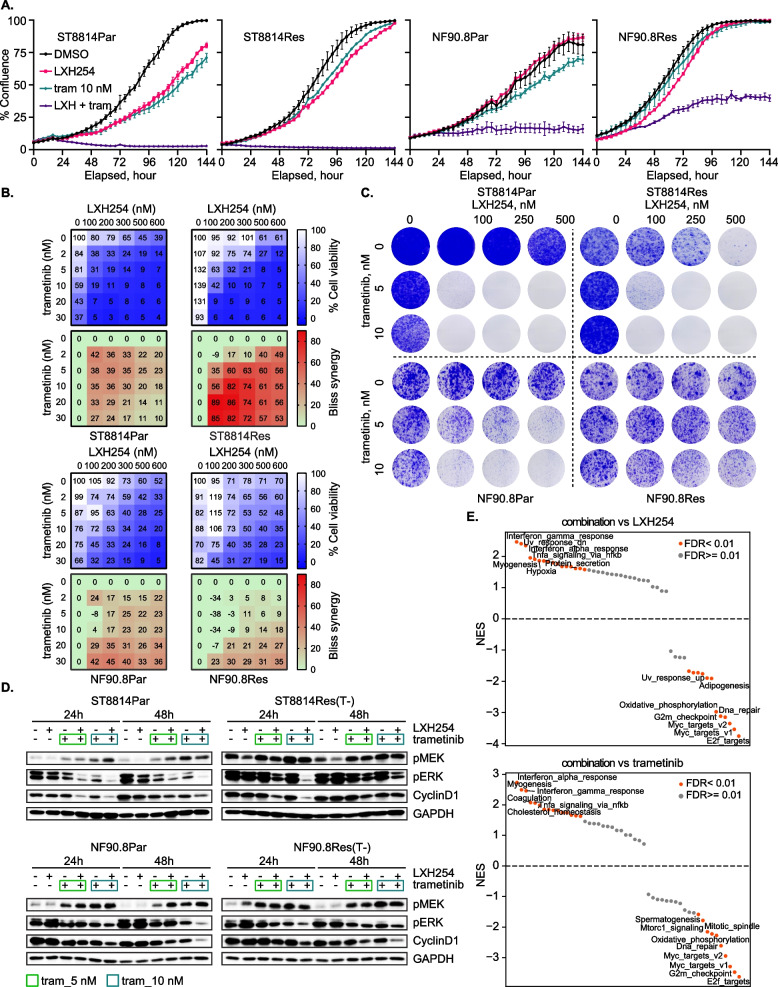
The combined inhibition of RAF and MEK is synergistic in MEKi-resistant models. **A** ST8814 and NF90.8 parental (Par) and trametinib-resistant (Res) cells were treated with DMSO, 250 nM LXH254, 10 nM trametinib or their combination, and cell confluence was monitored using IncuCyte. Shown are representative experiments from three biological replicates with three technical replicates in each setup **B** ST8814 and NF90.8 parental (Par) and trametinib-resistant (Res) cells were treated with DMSO, increasing concentrations of LXH254 and/or trametinib for 5 days. Cell viability was determined using MTT assay and bliss synergy scores were calculated using Combenefit software. Shown are representative experiments from two biological replicates with three technical replicates in each setup. **C** Cell lines as in A. were treated with DMSO, increasing concentrations of LXH254 and/or trametinib for two weeks. Cell growth was assessed using crystal violet staining. Shown are representative images from two (ST8814 pairs) and four (NF90.8 pairs) biological replicates. **D** Cell lines as in A. were treated as described in Fig. 4D. for 24 and 48 h and the indicated proteins were detected using immunoblot. Shown are representative images from three biological replicates. **E** ST8814, NF90.8 and S462 cells were treated with DMSO, 250 nM LXH254 and/or 10 nM trametinib for 24 h. GSEA of bulk RNAseq results revealed significantly (FDR < 0.01) enriched gene sets for the combination as compared to single agent treatments. Shown is the cancer hallmark pathway distribution (n = 50) between LXH254 + trametinib combination and LXH254 (top) or trametinib (bottom) treatment. NES were calculated by GSEA. Red dot represents a pathway with FDR < 0.01, and the pathways with FDR < 0.01 are listed. NES = normalized enrichment scores. FDR = false discovery rate

We therefore hypothesized that upregulation of RTK activity in part converged on CRAF for reactivation of ERK signaling, leading to MEKi resistance, and type 2 RAFi plus MEKi might be effective at overcoming MEKi resistance. Considering this, we assessed the efficacy of this combination on cell growth and observed synergistic inhibition in the two parental/ resistant pairs (Figs. 5A-C, S5A). These results prompted us to test the effects of combined inhibition of RAF and MEK on ERK signaling. We treated parental/ resistant cells with DMSO, LXH254, trametinib or their combination over a 48-h time course. Combined treatment with LXH254 and trametinib demonstrated greater suppression of p-ERK and cyclin D1, relative to either compound alone (Figs. 5D, and S5B).

To further explore mechanisms contributing to sensitivity to LXH254 plus trametinib, we performed bulk RNA sequencing (RNAseq) on ST8814, NF90.8 and S462 cells (Fig. 5E and Table S2). When comparing the combination with either single agent, LXH254 or trametinib, we found significant (False Discovery Rate, FDR < 0.01) negative enrichment of cancer hallmark gene sets such as Myc targets, cell cycle (E2F targets, G2/M checkpoint), DNA repair and oxidative phosphorylation. These results implied that LXH254 plus trametinib more potently suppressed ERK signaling, cell proliferation and cell cycle, DNA repair, as well as metabolic oxidative phosphorylation to become active against cancer cell growth.

### Combined inhibition of RAF and MEK demonstrates efficacy in in vivo cell line and patient-derived models of NF1-MPNST

**Fig. 6:**
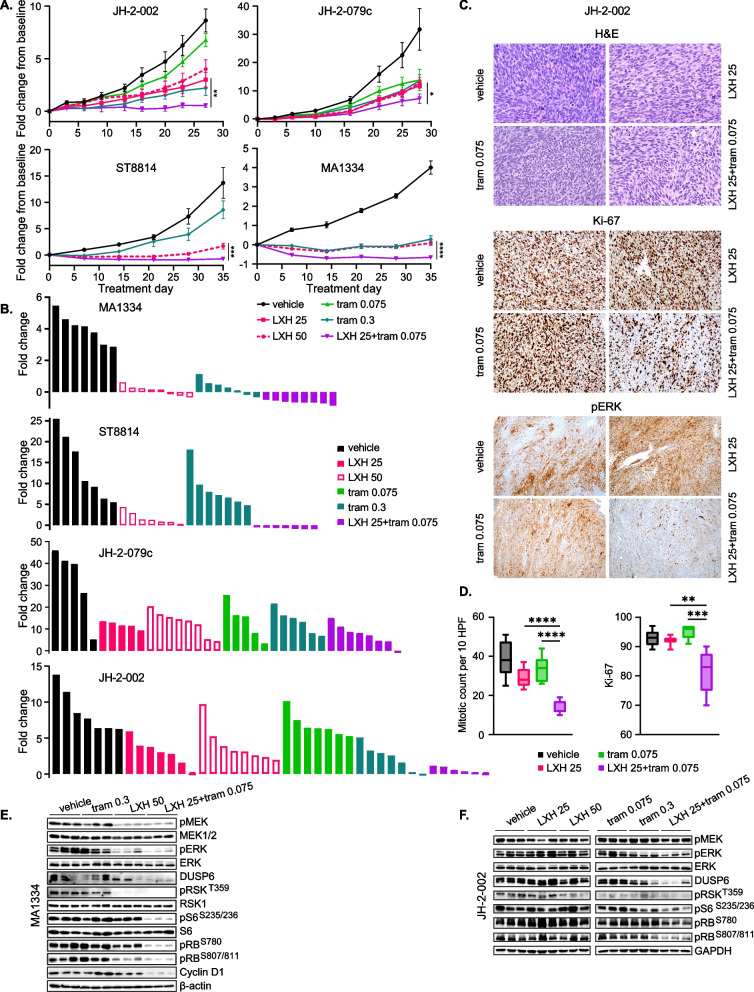
Combined inhibition of RAF and MEK demonstrates efficacy in in vivo cell line and patient-derived models of NF1-MPNST. **A** Three NF1-MPNST patient-derived (JH-2–002, JH-2-079c and MA1334) and one cell-line-derived (ST8814) xenograft models were treated with vehicle, LXH254 (25 and 50 mg/kg, BID-twice daily), trametinib (0.075 and 0.3 mg/kg, QD-once daily) and their combination (dose as shown) for 4–5 weeks (n = 7–8 mice/group). Statistical significance was analyzed between LXH254 and the combination groups at the endpoint, *P* < 0.05, *; *P* < 0.01, **; *P* < 0.001, ***; *P* < 0.0001, ****. **B** Waterfall plots representing each individual fold change in tumor growth [end point versus day 0, (day X/ day 0)-1] are shown. Some mice with tumor size or other conditions reaching the protocol limit were removed from the study before the endpoint, and therefore their tumor volume values at the endpoint were not available in data presentation. **C**-**D** Three tumors per arm from PDX JH-2–002 as shown in **B** were randomly selected for hematoxylin and eosin stain, IHC for Ki-67, p-ERK (**C**). Quantification of mitotic counts and Ki-67 was performed through blind evaluation using QuPath. p-ERK signal was assessed as “extensive” for the vehicle, LXH254 and trametinib arms, and “minimal” for the combination arm. HPF = high-power fields (**D**). **E**-**F** Immunoblotting assessment for ERK signaling activity in PDX MA1334 treated with drugs for 3 days (**E**) and in JH-2–002 on therapy for 4 weeks using regimens as in **A**. (**F**) Tumors were collected 4 h post last dose (**C**-**F**)

Based on these data, we hypothesized that in NF1-deficient MPNST, vertical inhibition of ERK signaling by pan-RAFi plus MEKi would enhance the efficacy of single-agent MEKi through partially overcoming both the adaptive and acquired resistance to MEKi. LXH254 has exhibited an acceptable safety profile, pharmacodynamic activity, and limited anti-tumor activity as a single agent in patients with advanced solid tumors driven by ERK signaling alterations, necessitating combination therapies to improve its efficacy (Janku et al. 2024). We thus tested the effects of combined LXH254 and trametinib in four xenograft models of NF1-MPNST, including three patient-derived (JH-2–002, JH-2-079c and MA1334) and one cell-line derived (ST8814). Continuous treatment dosing over 4–5 weeks, compared to either single agent was used (Fig. 6A-B and Table S3). A notable benefit of the combination in reducing tumor growth was observed in three models, JH-2–002, ST8814 and MA1334, and additive anti-tumor activity was seen in JH-2-079c (Fig. 6A-B). We visualized the fold-change growth of individual tumors using waterfall plots by analyzing the ratio of changes in tumor volume versus baseline, where enhanced anti-tumor activity in mice treated with the combination was also seen (Fig. 6B). The combination of LXH254 plus trametinib was dosed at 25 mg/kg BID and 0.075 mg/kg QD, respectively, to be in the range of the two clinically tolerated combination schemes identified for this combination (Braud et al. 2023). The clinically relevant doses of each single agent were included for comparison. In PDX JH-2–002, the combination significantly repressed tumor mitotic activity and proliferation as indicated by mitotic counts and Ki-67 decrease (Fig. 6C-D) and markedly inhibited p-ERK, DUSP6 and several other downstream effectors (p-RSK, p-S6, p-RB, Cyclin D1) (Figs. 6C, 6E, 6F, S6A-C). Overall, the combination of LXH254 plus trametinib demonstrated overall better anti-tumor activity in our four xenograft models of NF1-MPNST through greater inhibition in ERK and cell cycle signaling, and subsequent reduction in tumor proliferation.

## Discussion

Tumors with loss of NF1, both benign tumors such as pNF, and high-grade malignancies including MPNST, are characterized by hyperactivation of RAS/RAF/MEK/ERK signaling. Small molecule inhibitors targeting MEK (selumetinib) (Gross et al. 2020) and multiple tyrosine kinases (cabozantinib) (Fisher et al. 2021) have demonstrated clinical activity in tumor volume reduction and pain relief in patients with NF1-associated pNF. However monotherapy with these agents has demonstrated limited activity in models of MPNST (Wang et al. 2021), due to additional genomic alterations that drive disease progression from pNF to MPNST, such as loss of *CDKN2A* and PRC2 components *SUZ12* or *EED* (Lemberg et al. 2020). No clinical trials, however, have yet proven successful in treating patients with advanced MPNST (Kim and Pratilas 2018), and there is therefore an unmet need to advance preclinical studies of signaling networks in models of MPNST in order to develop effective strategies with novel agents. We previously have generated in vitro models of MPNST with acquired resistance to MEKi, in which upregulation of RTK activity (MET and PDGFRß) confers resistance. Based on these findings, several combinations of agents emerge as putative targets for translation to the clinic – including MEK and MET or MEK and SHP2 (Wang et al. 2021; Wang et al. 2020). Given the increasing number of patients with MPNST previously treated with MEKi for benign NF1-associated pNF and overlapping toxicity of co-targeting MEK and SHP2, a rationally designed alternate strategy involves targeting upstream RAS nucleotide cycling together with downstream effectors of ERK signaling. This concept led to the demonstration of the preclinical efficacy of combined SHP2 and CDK4/6 inhibition (Wang et al. 2023). Likewise, considering the inability to directly target NF1 loss, current research efforts are directed toward reducing the accumulation of RAS-GTP as a result of loss of NF1. Thus, targeting RAS guanine exchange factor complex (SOS1/2-SHP2-GAB1-GRB2), becomes a critical therapeutic strategy in NF1-null tumors (Wang et al. 2020; Wang et al. 2023; Jackson et al. 2023).

Here, we demonstrate the inhibitory effects of the NF1 GAP-related domain on RAS effector signaling and cell growth of NF1-MPNST, which is consistent with previous reports (Bai et al. 2019). We further support the sensitizing role of NF1 GAP function in response to MEKi, as re-expression of GRD in NF1-MPNST reduces RAS-GTP that is otherwise elevated by MEK inhibition, resulting in more durable ERK signaling inhibition. These data provide evidence for a combination regimen of NF1-GRD directed gene therapy with MEK targeted therapeutics in NF1-related tumors.

In this report, we interrogate the functional role of the three RAF paralogs – ARAF/ BRAF/ CRAF – in NF1-null MPNST. CRAF genetic depletion most markedly reduced cell growth and proliferation in MPNST with loss of NF1, followed by moderate reduction with BRAF knockdown, and only minimal effect with ARAF suppression. Concomitant ablation of CRAF and BRAF provided further inhibition of cell growth, indicative of the driver role of CRAF/BRAF in ERK signal transduction driven by loss of NF1, in line with a recent report highlighting that MEKi-induced CRAF/BRAF dimer formation confers resistance to MEKi (Miranda-Roman et al. 2024). We found that NF1-MPNST cells are relatively sensitive to the type 2 RAFi LXH254 (naporafenib), and further that baseline ARAF protein level was not inversely associated with sensitivity to LXH254. These data suggested that despite being dispensable in a native state, ARAF may adaptively relay signal from RAS to MEK to serve as a rescue mechanism after blockade of CRAF/BRAF kinase activity by type 2 RAFi and therefore may mediate resistance to this class of agents. This concept aligns with two recent reports. The first report demonstrated that ARAF overexpression reduces sensitivity to LXH254 in *K/NRAS*-mutant cancers (Monaco et al. 2021). The second report showed that ARAF activating mutations confer both preclinical and clinical resistance to belvarafenib monotherapy in melanoma (Yen et al. 2021), where the resistance is dependent on kinase activity and dimer formation. Consequently, these findings warrant a strategy combining RAF dimer inhibitors with agents targeting downstream of ARAF, such as MEKi.

In addition to NF1 GAP function, CRAF/BRAF genetic abrogation also enhances the sensitivity of NF1-MPNST cells to MEKi through sustained inhibition of ERK signaling, providing the rationale for combined type 2 RAFi with MEKi in this type of disease. Reported knockdown screens have identified a synthetic lethal interaction between CRAF genetic ablation and MEK inhibition in *KRAS*-mutant cancers (Lamba et al. 2014). Another genome-wide RNA interference screen implicates NF1 loss in conferring resistance to type 1 RAFi in *BRAF* V600E-mutant cancers, while cancer cells harboring *BRAF* V600E and NF1 loss retain sensitivity to type 2 RAFi and ERKi, further suggesting the activity of vertical inhibition of ERK signaling with combination regimen consisting of type 2 RAFi in NF1 loss tumors (Whittaker et al. 2013). Here, we provide extensive evidence showing the preclinical anti-proliferative and anti-tumor activity of LXH254 plus trametinib, through more durable inhibition of ERK pathway, relative to either single agent, in a large panel of traditional and patient-derived models of NF1-MPNST, in vitro and in vivo, as well as in MEKi-resistant models driven by upregulation of RTK activity. Moreover, NF1 WT human Schwann cells are not sensitive to this combination, suggesting the NF1 loss tumor cells are uniquely responsive to simultaneous targeting of RAF dimerization and MEK, and the central role of ERK signaling in driving NF1-MPNST. We also examined the therapeutic benefits from combining LXH254 with type 1 RAFi including vemurafenib and dabrafenib and compared those to LXH254 plus trametinib. Type 2 RAFi plus MEKi demonstrated greater potency than type 2 plus type 1 RAFi. Our study demonstrates consistency with previous research (Whittaker et al. 2013) that NF1 loss mediates resistance to type 1 RAFi, in addition to the paradoxical activation of ERK signaling by type 1 RAFi in RAF WT cells. Investigation into this mechanism identified elevation of CRAF protein and activity in MEKi acquired resistant models driven by RTK activation, as well as in corresponding parentals after signaling adaptation to short-term exposure of MEKi. These results indicate that type 2 RAFi improves response to MEKi via suppression of CRAF/BRAF kinase activity induced by MEKi as a consequence of loss of negative feedback, producing prolonged inhibition of ERK and downstream cell cycle signaling and thereby achieving combination therapeutic benefit in NF1-null tumors.

Combined inhibition of RAF dimers and MEK demonstrates tumor regression through activity in cell growth signaling and the tumor immune microenvironment (TIME). A previous study reported the combinatorial efficacy of type 2 RAFi plus MEKi in both immunocompromised and immunocompetent models through tumor cell-intrinsic and extrinsic mechanisms. In this work, type 2 RAFi plus MEKi elicits anti-tumor activity via durable suppression of ERK signaling and immunologically induction of CD8 + T cells (Hong et al. 2021). Our RNAseq analysis revealed that compared to either single agent, LXH254 plus trametinib significantly (FDR < 0.01) downregulated the well-established and potential oncogenic events including cell proliferation (Myc targets), cell cycle (E2F targets and G2M checkpoint), DNA repair as well as energy metabolism oxidative phosphorylation, and significantly (FDR < 0.01) upregulated the tumor-suppressing events― interferon alpha and gamma response― all of which may contribute to mechanisms of sensitivity to this combinatorial approach. As genes related to E2F targets and G2M checkpoint were also identified as significant positively-enriched pathways in an analysis on MPNST versus pNF (Banerjee et al. 2024), their downregulation with this drug combination suggests effective interruptive of biological pathways driving malignant transformation. Mitochondrial oxidative phosphorylation has recently become an emerging target for cancer treatment, as several types of cancer depend on the metabolite ATP for survival, especially when under stress from chemo- or targeted- therapies. Oxidative phosphorylation is therefore also a resistance mechanism to cancer therapy (Ashton et al. 2018). Moreover, oxidative phosphorylation is associated with immune cell activation in the tumor immune microenvironment. Its inhibition overcomes tumor hypoxia, resulting in reactivation of immune response and induction of anti-tumor immunity (Boreel et al. 2021). Further, interferon alpha and gamma both have anti-proliferative, pro-apoptosis and cytotoxic effects directly on tumor cells, as well as indirectly contribute to anti-tumor immunity by affecting host cells including CD8 + T cells (Shi et al. 2022; Han et al. 2023), suggesting that tumor cell eradication by the combination type 2 RAFi plus MEKi is in part through the immunoediting of interferons. While our study did not examine cell death pathways and is limited to examining the efficacy of the combination in only immune-deficient models, cell death pathways and immune-modulating effects may also provide potential mechanisms associated with therapy response in addition to durable ERK signaling inhibition, and these effects remain to be studied or seen in human trials.

Numerous molecularly targeted therapies, which appeared highly effective in murine Nf1-MPNST preclinical studies, have proven ineffective when translated to human clinical trials. In order to faithfully represent the molecularly heterogeneous human MPNST, we utilized a combination of traditional and patient-derived cell line and xenograft models developed in our laboratory and relied on a foundation of molecular features and their correlation with responses to targeted therapy (Wang et al. 2021; Wang et al. 2023) to assess the efficacy of combined use of type 2 RAFi and MEKi. Overall, our findings, justified by rigorous in vitro evidence and consolidated with in vivo efficacy testing in patient-derived models, are mechanism-based and rationally designed. As naporafenib advances through stages of clinical development, there may be an accelerated potential for clinical translation, which provides treatment options for a broad spectrum of patients with diverse NF1 indications, including MPNST.

## Conclusions

Oncogenic RAS signaling pathways have been extensively studied in the context of RAS mutations in epithelial cancers, however little is known about RAS signaling in the context of NF1 loss in MPNST. We demonstrate for the first time that CRAF and BRAF, but not ARAF mediate RAS dependency in NF1-inactivated MPNST, and that paralog selective RAFi attenuates adaptive response to MEK inhibition. Aligned with this, we previously reported the genomic amplification of *RAF1* in MEKi-resistant MPNST models where transcriptional upregulation of PDGFRß drives acquired resistance (Wang et al. 2021). These findings were corroborated by Chi and colleagues, demonstrating that PDGFRß-driven MEKi resistance converges on promoting BRAF/CRAF dimerization (Miranda-Roman et al. 2024). Collectively, our study provides compelling evidence for the use of new class paralog-selective RAFi in NF1-MPNST and offers strong preclinical data that support their clinical application in combination with a MEKi.

## Supplementary Information

Supplementary Material 1.

Supplementary Material 2.

Supplementary Material 3.

Supplementary Material 4.

## Data Availability

RNA sequencing data was deposited in Gene Expression Omnibus with accession number GSE303418. All the other data generated in this study are available within the article and its Supplementary Data. Materials in this study are available upon a reasonable written request to the corresponding authors.

## Abbreviations

ATP: Adenosine triphosphate
BID: Bis in die
CAAX: Cysteine-Aliphatic Amino Acid-Aliphatic Amino Acid-X
CCK-8: Cell Counting Kit-8
CD8: Cluster of differentiation 8
CRISPR-CAS9: Clustered regularly interspaced short palindromic repeats and CRISPR-associated protein 9
DMSO: Dimethyl sulfoxide
DNA: Deoxyribonucleic acid
Dox: Doxycycline
FDA: Food and Drug Administration
FDR: False Discovery Rate
GAP: GTPase-activating protein
GI50: Growth inhibition 50%
GRD: GAP-related domain
GTP: Guanosine triphosphate
HPF: High-power fields
IACUC: Institutional Animal Care and Use Committee
IHC: Immunohistochemistry
IRB: Institutional Review Board
JH: Johns Hopkins
MEKi: MEK inhibitor
mg/kg: Milligrams per kilogram
MPNST: Malignant peripheral nerve sheath tumors
NBF: Neutral buffered formalin
NF1: Neurofibromatosis type 1
NF1: Neurofibromin
ng/mL: Nanograms per milliliter
nM: Nanomolar
PDX: Patient-derived xenograft
pNF: Plexiform neurofibroma
PRC2: Polycomb Repressive Complex 2
QD: Quaque die
RAFi: RAF inhibitor
Res: Resistant
RNAseq: RNA sequencing
RTK: Receptor tyrosine kinases
sgRNA: Single guide ribonucleic acid
shRNA: Short hairpin RNA
µM: Micromolar
US: United States
WT: Wild type
