# Supplementary material for: Selective inhibition of BRAF and CRAF sensitizes NF1-deficient malignant peripheral nerve sheath tumors to MEK inhibitors
Source: Mol Med. 2025 Sep 29;31:303. doi: 10.1186/s10020-025-01353-9 (PMC12482338; doi:10.1186/s10020-025-01353-9)
Supplement: Supplementary file 4 — Supplementary Material 4. [file 10020_2025_1353_MOESM4_ESM.pdf]

Supplementary Materials

Title: Selective inhibition of BRAF and CRAF sensitizes NF1-deficient malignant peripheral nerve sheath tumors to MEK inhibitors

Authors: Jiawan Wang[1], Arnab Sarkar[2,3], Natalia Garcia[2], Lindy Zhang[1], Ana Calizo[1], Alla Lisok[1], Katia Campos[2,3], Funan He[2], Nishanth Punjaala[2], Teresa Marple[2], Kai Pollard[1], Siyuan Zheng[2], Calixto-Hope Lucas[4], Vesselina G. Cooke[5], Christine A. Pratilas[1]*, Angelina V. Vaseva[2,3]*

Supplementary Methods

Cell lines, antibodies, and reagents

Five patient-derived NF1-MPNST cell lines (JH-2-002, JH-2-031, JH-2-055b, JH-2-079c and JH-2-103) (1) and two patient-derived xenografts (PDX, JH-2-002, JH-2-079c) were generated in our laboratories at Johns Hopkins (JH; Baltimore, MD, USA) from biospecimens collected during surgical resection from patients with NF1 (2). Material was collected under the Institutional Review Board–approved protocol (#J1649). All patients provided written informed consent. MA1334 xenograft (719797-321-R tumor model) was obtained from National Cancer Institute Patient-Derived Models Repository (NCI PDMR; Frederick, MD, USA; https://pdmr.cancer.govPDMR). STS26T, ST8814 and NF90.8 cell lines were from Dr. Gregory Riggins (JH; Baltimore, MD, USA). S462 cell line was provided by Dr. Peter Houghton (UT Health San Antonio, TX, USA). NF 94.3, NF96.2, NF10.1, NF11.1, ipn02.3-2λ, and ipn97.4 were provided by Dr. Margaret Wallace (University of Florida; Gainesville, FL, USA). All cell lines used in these experiments were verified by short-tandem repeat (STR) profiling for cell line authentication at Johns Hopkins University Core Facility or University of Texas, San Antonio, tested negative for mycoplasma contamination, and passaged *in vitro* for fewer than 3 months after resuscitation. All growth media were supplemented with 10% FBS, 2 mmol/L L-glutamine, and 1% penicillin–streptomycin. Trametinib-resistant cell lines were maintained in complete growth medium plus 20 nmol/L of trametinib (3).

LXH254 and trametinib were provided under a Materials Transfer Agreement with Novartis Biomedical Research (NBR; Cambridge, MA, USA). Dabrafenib and vemurafenib were purchased from MedChemExpress (Monmouth Junction, NJ, USA). Drugs for *in vitro* studies were dissolved in DMSO to yield 10 or 1 mmol/L stock solutions and stored at −20°C. For *in vivo* studies, trametinib was formulated in 0.5% hydroxypropyl methyl cellulose and 0.2% Tween 80 in water, pH 8; and LXH254 was formulated in MEPC4 vehicle (45% Cremophor RH40 + 27% PEG400 + 18% Corn Oil Glycerides (Maisine CC) + 10% ethanol), diluted at 1:4 with de-ionized water prior to dosing, ref. (4).

Immunoblotting

Cells were disrupted on ice in RIPA (radio immunoprecipitation assay) lysis buffer. Protein concentration was determined with Pierce BCA protein assay kit (#23227, Thermo Fisher Scientific; Waltham, MA, USA). Equal amounts of protein were separated by SDS-PAGE, transferred to nitrocellulose membranes,

immunoblotted with specific primary and secondary antibodies, and detected by chemiluminescence with the ECL detection reagents, Immobilon Western chemiluminescent HRP substrate (#WBKLS0500, Millipore; Billerica, MA, USA), or Pierce ECL Western blotting substrate (#32106, Thermo Fisher Scientific). The membranes were imaged using ChemiDoc touch imaging system (Bio-Rad; Hercules, CA, USA).

Cell proliferation assay

Cells were seeded in 96-well plates at 2,000 cells per well. A dose range of the compound indicated was prepared by serial dilutions and then added to the dishes containing adherent cells. Cells were incubated with drug for the indicated time. Cell growth was quantitated using the Cell Counting Kit-8 (Dojindo; Rockville, MD, USA) or MTT (Sigma; St. Louis, MO, USA) assays. For each condition, three to four replicates of each concentration were measured. Relative survival in the presence of drugs was normalized to the untreated controls after background subtraction. Graphs and GI50 were generated and calculated using Prism 10 based on the average of at least three replicates. Bliss synergy scores were calculated using Combenefit software https://sourceforge.net/projects/combenefit/.

Incucyte cell proliferation assays

2,000 cells per well were plated in 96-well plates and treated with the indicated dose of drugs. Cell confluence was monitored by a microscope gantry that was connected to a network external controller hard drive that gathered and processed image data, using the IncuCyte Live-Cell Imaging System (Essen BioSciences; Ann Arbor, MI, USA), which provides a time-lapse and an automated in-incubator method for quantifying cell growth.

Active RAS pull-down assay

Cells were seeded in 10-cm dishes. The following day, the 70% to 80% confluent cells were collected, and GTP-bound RAS was quantified using active RAS detection kit from Thermo Fisher (#16117) according to the manufacturer's instructions.

Lentivirus-based doxycycline-inducible expression

The human *NF1-GRD* fused with RAS CAAX motif and HA tag was amplified from *HA-NF1-CAAX STD* on a mammalian expression vector provided by Frederick National Laboratory for Cancer Research (5), and sub-cloned into the pCW57.1 vector harboring a Tet-regulated promoter (a gift from David Root, Addgene plasmid # 41393; Watertown, MA, USA). The lentiviruses encoding the *NF1-GRD-CAAX-HA* and *GFP* were packaged in HEK293T cells. The medium containing virus was filtered with 0.45µm PVDF filters followed by incubation with the target cells for 12 hours in the presence of 8 µg/mL of polybrene (#TR-1003-G, Millipore). The target cells were cultured in virus-free medium for one day and then selected with puromycin (2 µg/ml) for three days.

Lentivirus-based shRNA/ sgRNA-mediated knockdown cells

pLKO.1 lentiviral shRNA vectors targeting *ARAF*, *BRAF* and *CRAF* were obtained from Dr. Deborah Morrison, NCI (6). pLKO.1 control shRNA vector targeting *GFP* was a gift from David Sabatini (Addgene #30323). STS26T cells were infected overnight with human *NF1* sgRNA CRISPR all-in-one lentivirus (pLenti-U6-sgRNA-SFFV-Cas9-2A-Puro as the backbone) purchased from Applied Biological Materials (Richmond, BC, Canada). The lentiviruses encoding sh*GFP* and sh*ARAF*/ *BRAF*/ *CRAF* were packaged in HEK293T cells. Virus multiplicity of infection (MOI) was determined as previously described (6). The target cells were infected overnight by filtered viral supernatant at MOI of 2 for single knockdown or 0.2 for simultaneous knockdown of BRAF and CRAF in the presence of 8 μg/mL of polybrene (#TR-1003-G, Millipore). The infected cells were selected with puromycin (1-2 μg/mL) for four days before the analysis of the knockdown effects.

Colony formation assay

NF1-MPNST cells were seeded at low density, then treated with DMSO, LXH254 (100, 250 and 500 nmol/L), and/or trametinib (5 and 10 nmol/L) for 2 weeks. Cells were washed with PBS, fixed with 10% neutral buffered formalin, and then stained with 0.1% crystal violet for 30 minutes.

RNA sequencing data processing and gene set enrichment analysis (GSEA)

RNA sequencing data were processed following a previous study (7). Trim Galore (8) was applied to raw sequencing data to remove the adapter and poor-quality reads. STAR (Spliced Transcripts Alignment to a Reference) was used to align RNA sequencing data to the reference genome. To remove mouse-derived reads in PDX, we mapped the sequencing data to the human (GRCh38, GENCODE v29) and mouse (GRCm38, GENCODE vM19) reference genomes (9). Disambiguate (10) was then employed on the BAM (Binary Alignment Map) files to remove mouse reads. RSEM (RNA-Seq by Expectation-Maximization) (11) was applied to calculate raw read counts. GSEA was applied to identify significantly different pathways between different drug combinations. Cancer hallmark gene sets were downloaded from MSigDB (12). GSEA analysis was done by python package GSEApy (13), and the plot was generated by python package matplotlib (14) and seaborn (15). Quality control metrics are shown in **Table S2**.

Immunohistochemistry (IHC)

Hematoxylin and eosin (H&E) stain, Ki-67 and p-ERK IHC were performed at the Oncology Tissue Services Core of Johns Hopkins University. Three tumors per treatment were randomly selected. Briefly, immunolabeling was performed on formalin-fixed, paraffin-embedded sections on a Ventana Discovery Ultra autostainer (Roche Diagnostics; Indianapolis, IN, USA). Following dewaxing and rehydration on board, epitope retrieval was performed using Ventana Ultra CC1 buffer (#6414575001, Roche Diagnostics) at 96°C for 48 (Ki-67) and 64 (p-ERK) minutes, respectively. Primary antibodies anti-Ki-67 (1:200 dilution; #Ab16667, lot number GR3185488-1, Abcam; Waltham, MA, USA) and anti-phospho-ERK1/2-Thr202/Tyr204 (1:250 dilution; #9101, Cell Signaling Technology; Danvers, MA, USA) were applied at 36°C for 60 minutes. Primary antibodies were detected using an anti-rabbit HQ detection system (#7017936001 and 7017812001, Roche Diagnostics) followed by Chromomap DAB IHC detection kit

(#5266645001, Roche Diagnostics), counterstaining with Mayer's hematoxylin, dehydration, and mounting. Whole slide scanning was carried out at up to 40X magnification (0.23 microns/pixel) using a Hamamatsu Nanozoomer S210 digital slide scanner (Hamamatsu Photonics; Shizuoka, Japan). Whole slide images were visualized in Concentriq digital pathology platform (Proscia; Philadelphia, PA, USA), and representative images were shown at the magnification of 40X for hematoxylin and eosin stain, 20X for Ki-67 and 10X for p-ERK. Mitotic activity is expressed as a mitotic count, a measure of the number of mitoses per 10 contiguous 40X fields. Quantification of the signal (3 counts/ tumor) was performed by a pathologist with blind evaluation using QuPath software.

Supplementary Figures and Legends

Figure S1

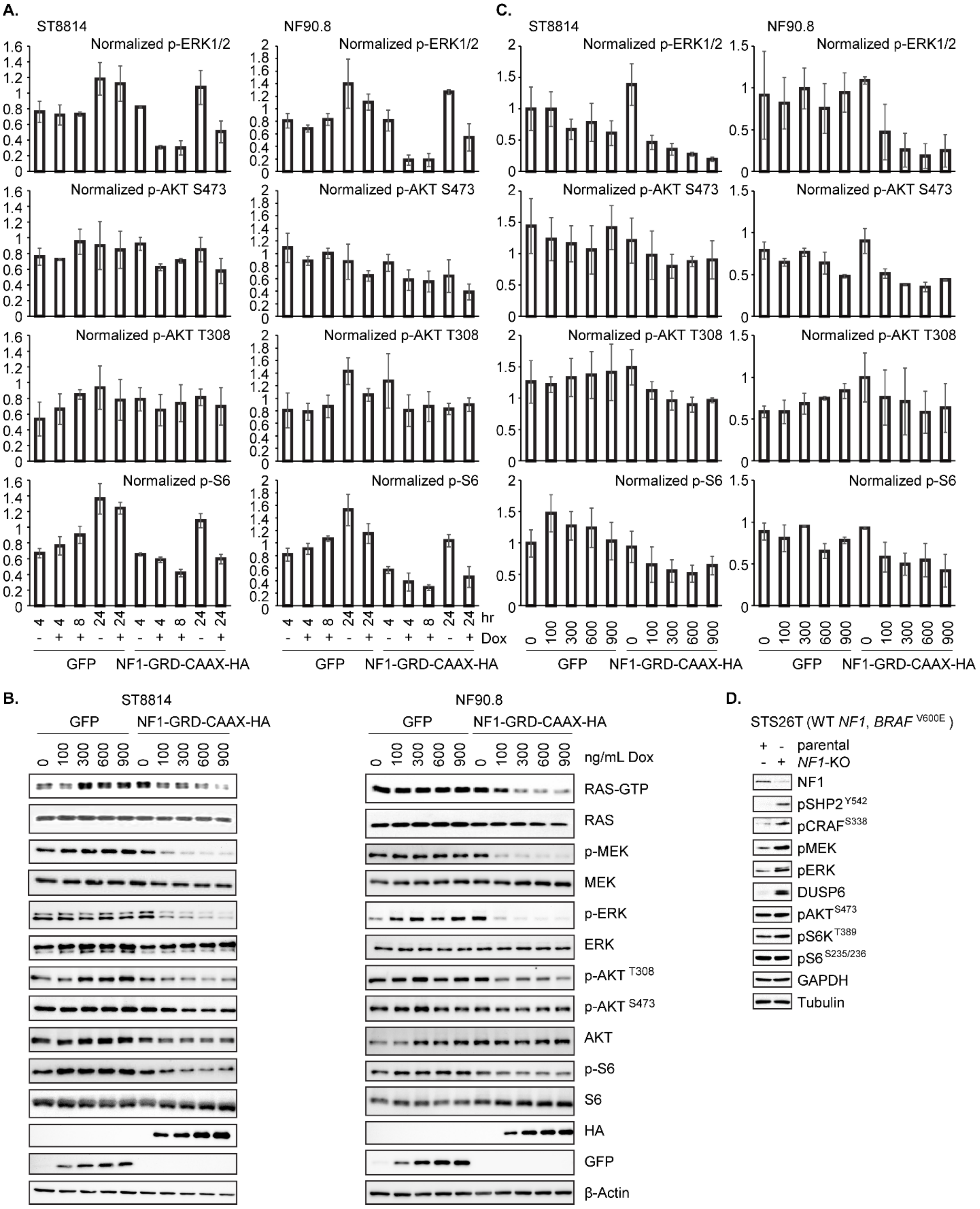

Figure S1. A. Bar graphs summarizing phospho-ERK1/2, phospho-AKT, and phospho-S6 protein levels normalized to actin levels in ST8814 and NF90.8 cells expressing doxycycline (Dox)-inducible GFP or NF1-GRD-CAAX. Data corresponds to Figure 1A. Protein levels were quantified from blots using ImageJ. Error bars represent SEM from three biological replicates. **B**. NF1-MPNST cells expressing Dox-inducible GFP or NF1-GRD-CAAX vector were treated for 8 hours with vehicle or Dox at the indicated concentrations and proteins were analyzed with western blotting. **C**. Bar graphs summarizing phospho-ERK1/2, phospho-AKT, and phospho-S6 protein levels normalized to actin levels. Data corresponds to Figure S1B. Protein levels were quantified from blots using ImageJ. Error bars represent SEM from three biological replicates. **D**. STS26T cells were infected overnight with human *NF1* sgRNA CRISPR all-in-one lentivirus. Signaling intermediates involved in RAS effector pathways (ERK and AKT/ mTOR) were detected using immunoblot. Parental= non-infected control.

Figure S2

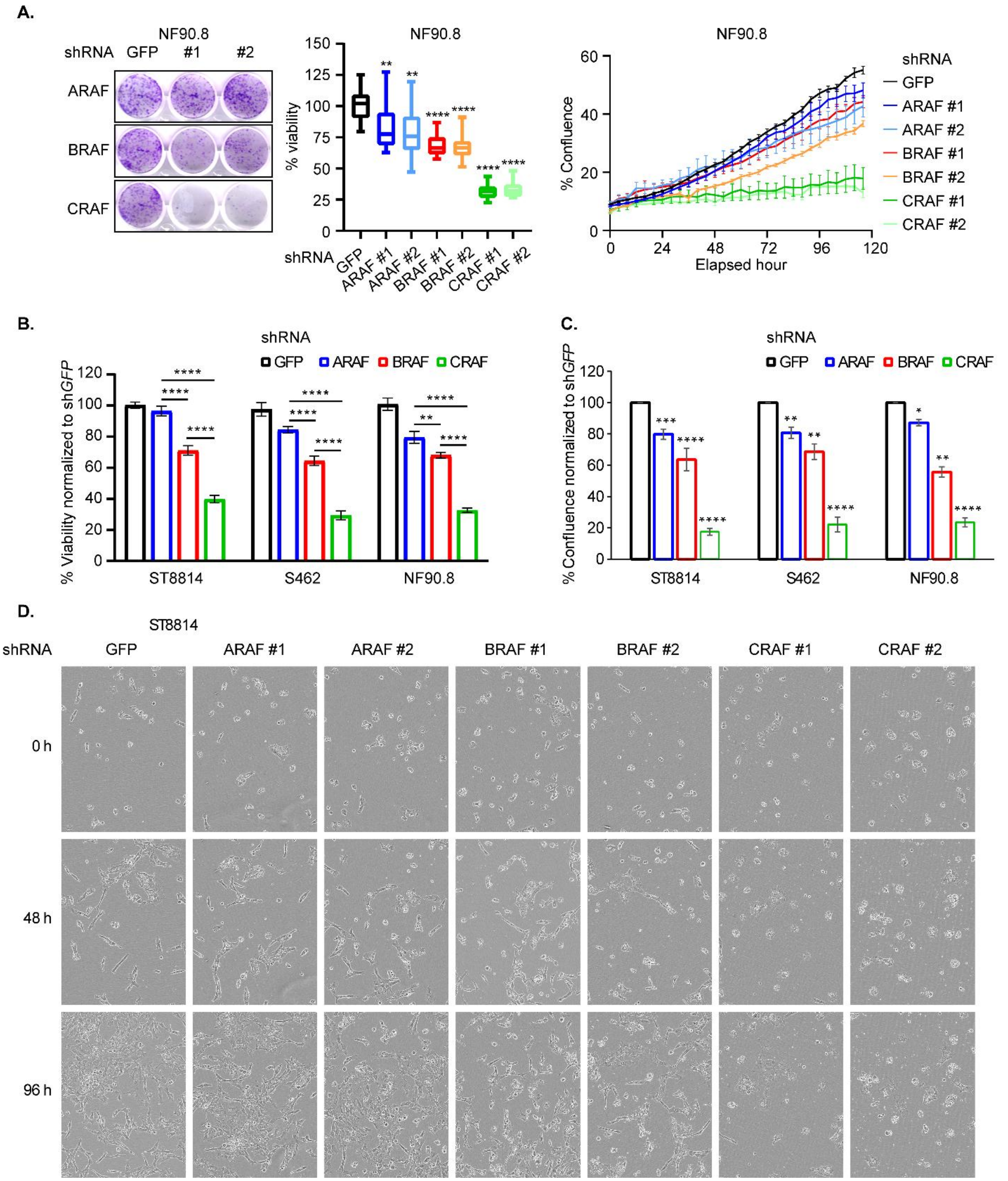
A.
NF90.8
shRNA GFP #1 #2
ARAF
BRAF
CRAF
% viability
shRNA GFP ARAF #1 ARAF #2 BRAF #1 BRAF #2 CRAF #1 CRAF #2
% Confluence
Elapsed hour
shRNA
GFP
ARAF #1
ARAF #2
BRAF #1
BRAF #2
CRAF #1
CRAF #2
B.
shRNA
GFP ARAF BRAF CRAF
% Viability normalized to shGFP
ST8814
S462
NF90.8
C.
shRNA
GFP ARAF BRAF CRAF
% Confluence normalized to shGFP
ST8814
S462
NF90.8
D.
ST8814
shRNA GFP ARAF #1 ARAF #2 BRAF #1 BRAF #2 CRAF #1 CRAF #2
0 h
48 h
96 h

Figure S2

E.

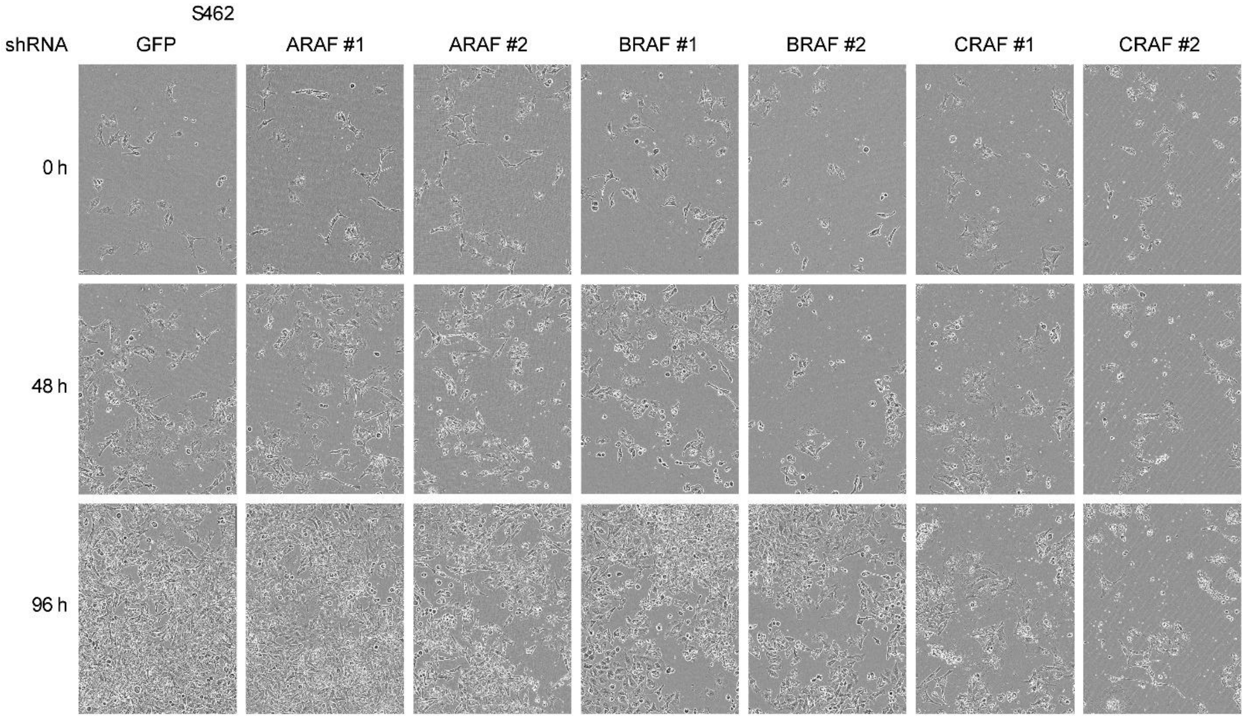
S462
shRNA
GFP
ARAF #1
ARAF #2
BRAF #1
BRAF #2
CRAF #1
CRAF #2
0 h
48 h
96 h

F.

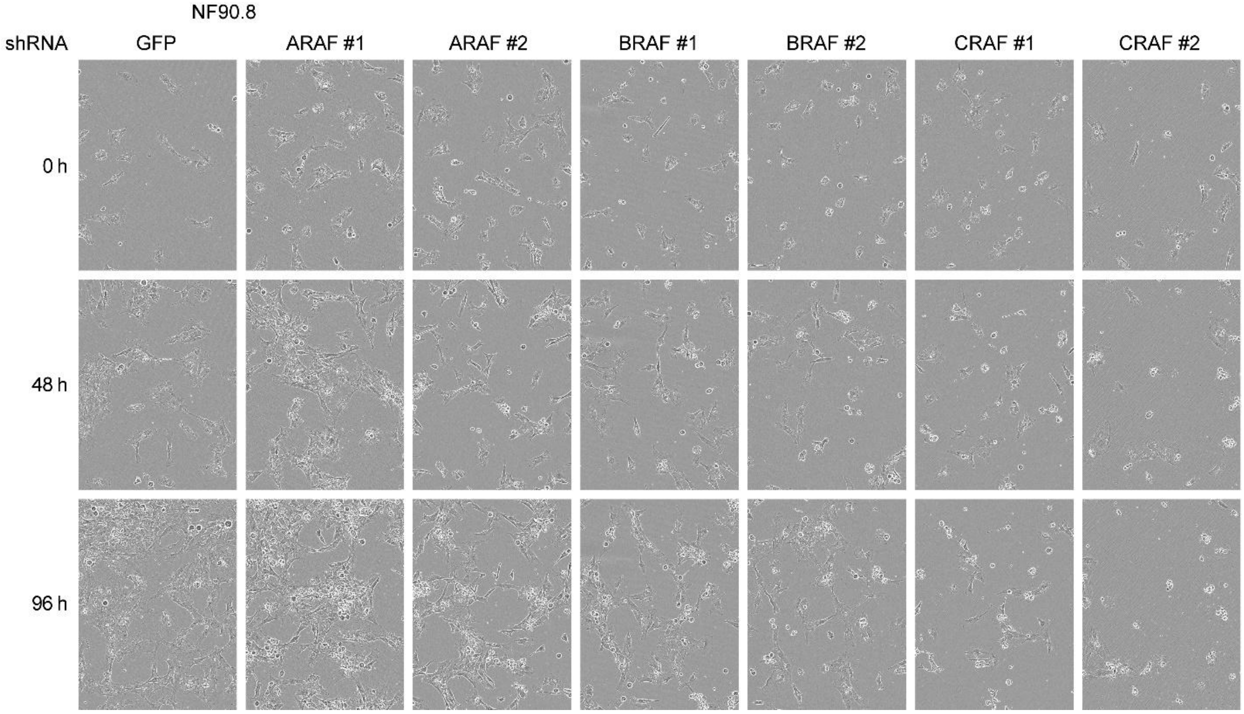
NF90.8
shRNA
GFP
ARAF #1
ARAF #2
BRAF #1
BRAF #2
CRAF #1
CRAF #2
0 h
48 h
96 h

Figure S2

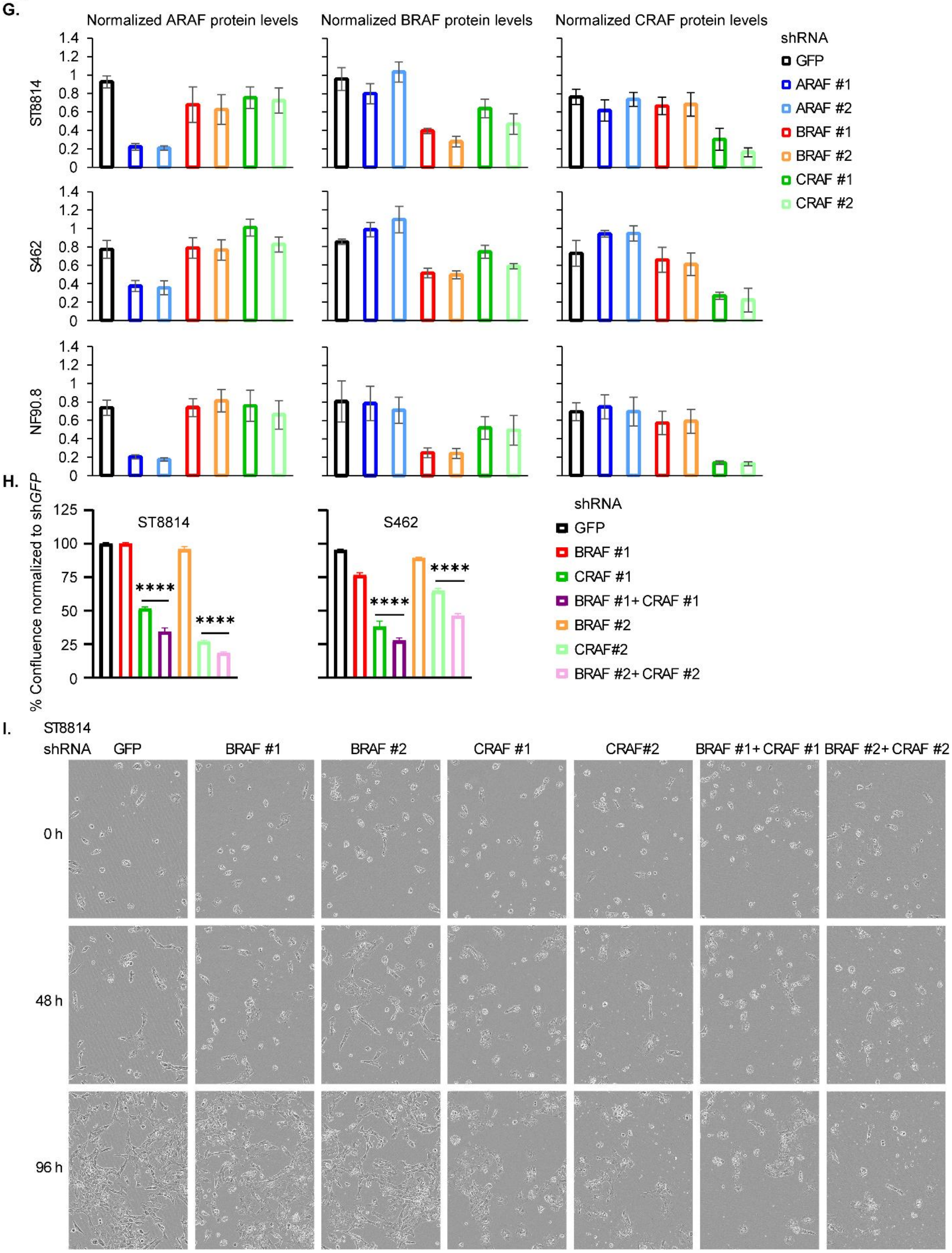
G.
Normalized ARAF protein levels
Normalized BRAF protein levels
Normalized CRAF protein levels
ST8814
S462
NF90.8
1.4
1.2
1
0.8
0.6
0.4
0.2
0
shRNA
GFP
ARAF #1
ARAF #2
BRAF #1
BRAF #2
CRAF #1
CRAF #2
H.
% Confluence normalized to shGFP
ST8814
S462
125
100
75
50
25
0
****
shRNA
GFP
BRAF #1
CRAF #1
BRAF #1+ CRAF #1
BRAF #2
CRAF#2
BRAF #2+ CRAF #2
I.
ST8814
shRNA GFP BRAF #1 BRAF #2 CRAF #1 CRAF#2 BRAF #1+ CRAF #1 BRAF #2+ CRAF #2
0 h
48 h
96 h

Figure S2

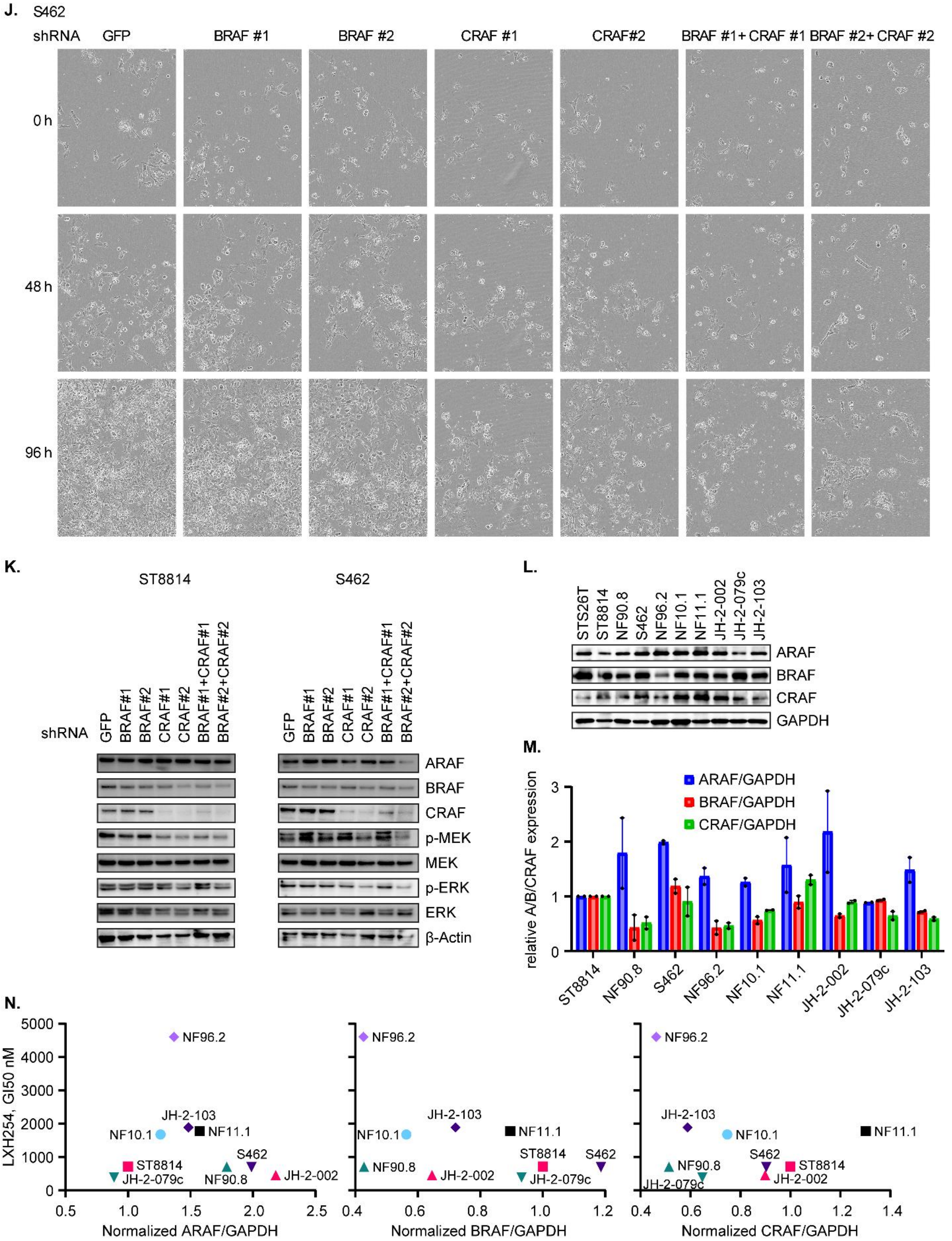
J.
S462
shRNA
GFP
BRAF #1
BRAF #2
CRAF #1
CRAF#2
BRAF #1+ CRAF #1
BRAF #2+ CRAF #2
0 h
48 h
96 h
K.
ST8814
S462
shRNA
GFP
BRAF#1
BRAF#2
CRAF#1
CRAF#2
BRAF#1+CRAF#1
BRAF#2+CRAF#2
ARAF
BRAF
CRAF
p-MEK
MEK
p-ERK
ERK
β-Actin
L.
STS26T
ST8814
NF90.8
S462
NF96.2
NF10.1
NF11.1
JH-2-002
JH-2-079c
JH-2-103
ARAF
BRAF
CRAF
GAPDH
M.
ARAF/GAPDH
BRAF/GAPDH
CRAF/GAPDH
relative A/B/CRAF expression
0
1
2
3
ST8814
NF90.8
S462
NF96.2
NF10.1
NF11.1
JH-2-002
JH-2-079c
JH-2-103
N.
LXH254, GI50 nM
0
1000
2000
3000
4000
5000
NF96.2
JH-2-103
NF10.1
NF11.1
ST8814
S462
NF90.8
JH-2-079c
JH-2-002
0.5
1.0
1.5
2.0
2.5
Normalized ARAF/GAPDH
0.4
0.6
0.8
1.0
1.2
Normalized BRAF/GAPDH
1.4
Normalized CRAF/GAPDH

Figure S2. **A**. Left: NF90.8 cells transduced with lentiviral vectors expressing shRNA targeting *GFP* or two independent sequences targeting *ARAF*, *BRAF* or *CRAF* were subject to crystal violet staining as described for ST8814 and S462 cells in Figure 2A left. Representative images from three biological replicates are shown; Middle: cell viability assay normalized to sh*GFP* expressing cells using MTT reagents. Cells were treated as described for ST8814 and S462 cells in Figure 2A middle. Error bars represent SEM from three biological replicates with three technical replicates in each setup. Each experimental group was compared to sh*GFP* group and statistical difference was determined using unpaired Student t-test with GraphPad Prism software. ns= not significant, * $p<0.05$, ** $p<0.01$, *** $p<0.001$, **** $p<0.0001$; Right: IncuCyte phase contrast imaging of cells treated as in Figure 2A right. Shown are representative experiments from three biological replicates with three technical replicates in each setup. **B**. Bar graphs summarizing percent viability of *ARAF*, *BRAF*, or *CRAF* shRNA-expressing NF1-MPNST cells normalized to sh*GFP*-expressing cells. Graphs correspond to Figures 2A middle and S2A middle. Error bars represent SEM from three biological replicates with three technical replicates in each setup. Experimental groups were compared to each other, and statistical difference was determined using unpaired t-test with GraphPad Prism software. **$p< 0.01$, **** $p<0.0001$. **C**. Bar graphs summarizing percent confluency derived from IncuCyte phase contrast imaging of NF1-MPNST cells expressing *ARAF*, *BRAF*, or *CRAF* shRNA normalized to sh*GFP*-expressing cells. Graphs correspond to Figures 2A right and S2A right. Cells were treated as in Figure 2A right, and percent confluency at day 5 was normalized to the confluency of sh*GFP* cells. Error bars represent SEM from three (S462 and NF90.8) or four (ST8814) biological replicates with three technical replicates in each setup. Each experimental group was compared to sh*GFP* group and statistical difference was determined using unpaired Student t-test with GraphPad Prism software. *$p<0.05$, **$p<0.01$, ***$p<0.001$, **** $p<0.0001$. **D-F**. Representative images from IncuCyte phase contrast imaging of ST8814 (D), S462 (E), and NF90.8 (F) cells expressing sh*GFP* control or *ARAF*, *BRAF*, or *CRAF* shRNAs. Images correspond to the data presented in Figures 2A right and S2A right. **G**. Bar graphs summarizing A/B/CRAF protein levels normalized to actin levels in NF1-MPNST cells following A/B/CRAF knockdown. Graphs correspond to Figure 2C. Protein bands were quantified from western blots using ImageJ. Error bars represent SEM from three biological replicates. **H**. Bar graphs summarizing percent confluency derived from IncuCyte phase contrast imaging of NF1-MPNST cells expressing *BRAF*, *CRAF*, or *BRAF*+*CRAF* shRNAs normalized to sh*GFP*-expressing cells. Graphs correspond to the data in Figure 2D. Cells were treated as in Figure 2D, and percent confluency at day 5 was normalized to the confluency of sh*GFP* cells. Error bars represent SEM from three technical replicates in one setup. Data is representative of two biological replicates. sh*CRAF* group was compared to sh*BRAF*+sh*CRAF* group and statistical difference was determined using unpaired Student t-test with GraphPad Prism software. **** $p<0.0001$. **I-J**. Representative images from IncuCyte phase contrast imaging of ST8814 (I) and S462 (J) cells expressing sh*GFP* control or *BRAF*, *CRAF*, or *BRAF*+*CRAF* shRNAs with reduced MOI. Images correspond to the data presented in Figure 2D. **K**. Cells as described in Figure 2D were analyzed for protein expression using immunoblot. **L**. Cells as described in Figure 2E were analyzed for isoform-specific RAF protein expression using immunoblot. Images are representative of two biological replicates. **M**. Bar graphs summarizing relative A/B/CRAF protein levels in NF1-MPNST cells. Graphs are related to Figure S2L. Protein bands were quantified using Image J and the relative RAF expression (the ratio of RAF/ GAPDH) was normalized to the expression in ST8814 cells. Error bars represent SEM from two biological replicates.

N. Correlation analysis between relative RAF expression and GI50 of LXH254 performed using GraphPad prism 10.

Figure S3

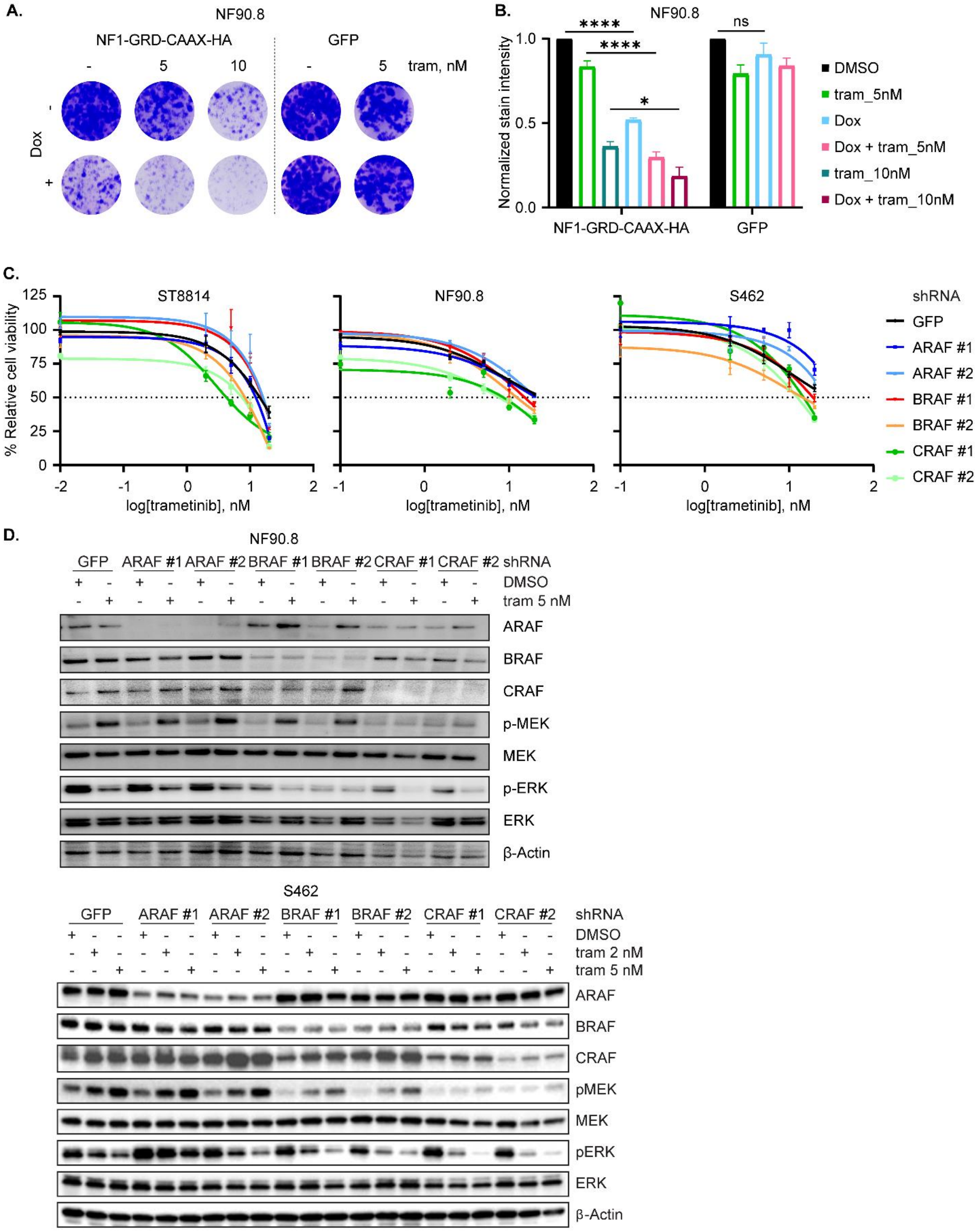

Figure S3. **A**. NF90.8 as described in Figure 1 was treated with DMSO, doxycycline (Dox, 300 ng/ml), trametinib (tram, 5 and 10 nM) or their combination for two weeks. Cell growth was evaluated using crystal violet staining. Representative images from two biological replicates are shown. **B**. Bar graph summarizing quantification of crystal violet staining of NF90.8 cells expressing Dox-inducible *GFP* or *NF1-GRD* vector and treated with trametinib. Graphs correspond to Figure S3A. Error bars represent SEM from two biological replicates with three technical replicates in each setup, and statistical difference was determined using unpaired t-test with GraphPad Prism software. *p< 0.05, **** p<0.0001, ns= not significant. **C**. Normalized dose response curves of NF1-MPNST cells treated with increasing concentrations of trametinib. Graphs are related to Figures 3B-C. Shown are representative graphs from three biological replicates with three technical replicates in each setup. **D**. NF90.8 and S462 as described in Figures 2A and S2A were treated with DMSO or trametinib (tram, 2 and 5 nM) for 24 hours. Proteins were detected using immunoblots. Representative images from three (NF90.8) or five (S462) biological replicates are shown.

Figure S4

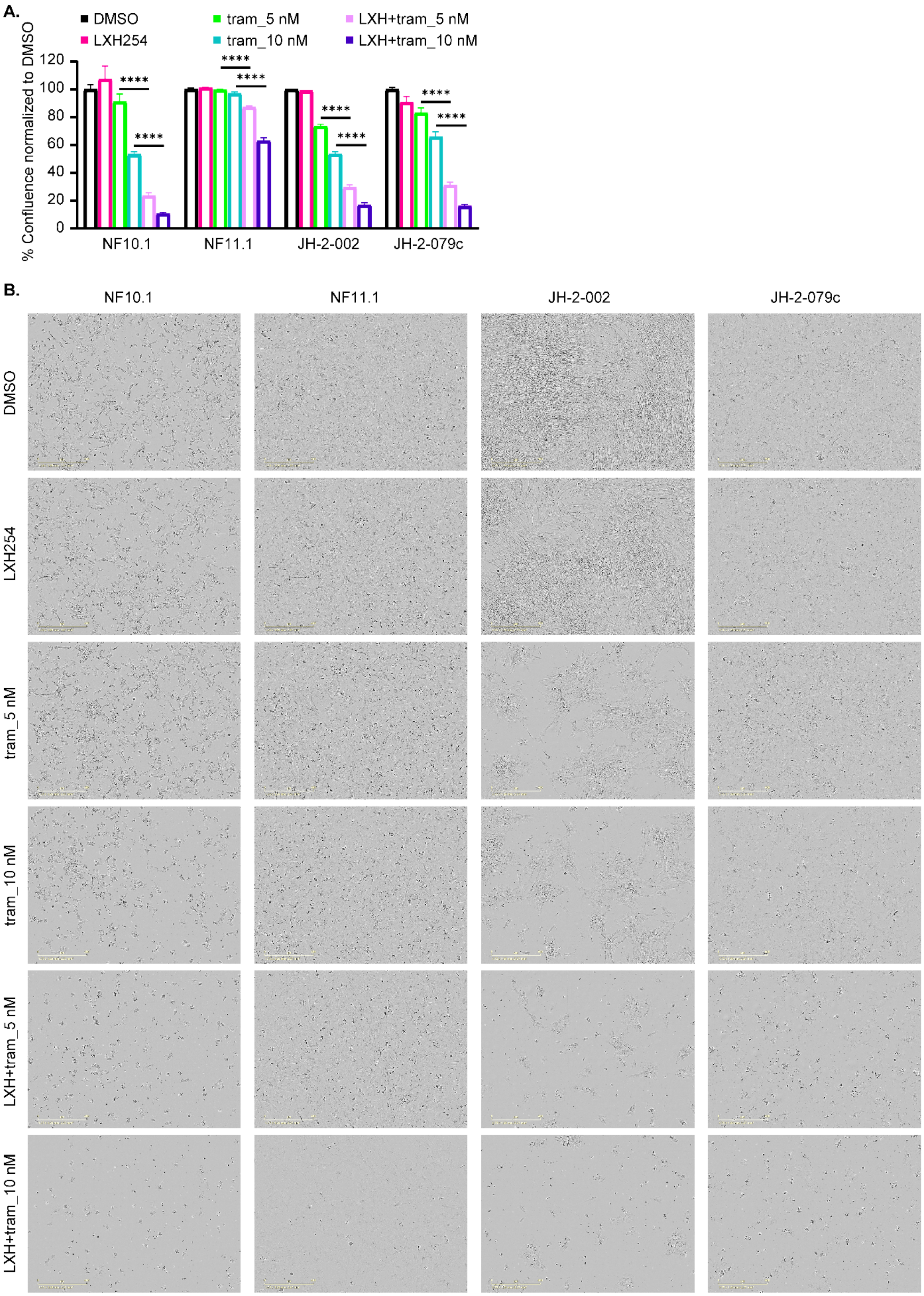
A.
DMSO
LXH254
tram_5 nM
tram_10 nM
LXH+tram_5 nM
LXH+tram_10 nM
% Confluence normalized to DMSO
120
100
80
60
40
20
0
****
NF10.1
NF11.1
JH-2-002
JH-2-079c
B.
NF10.1
NF11.1
JH-2-002
JH-2-079c
DMSO
LXH254
tram_5 nM
tram_10 nM
LXH+tram_5 nM
LXH+tram_10 nM

Figure S4

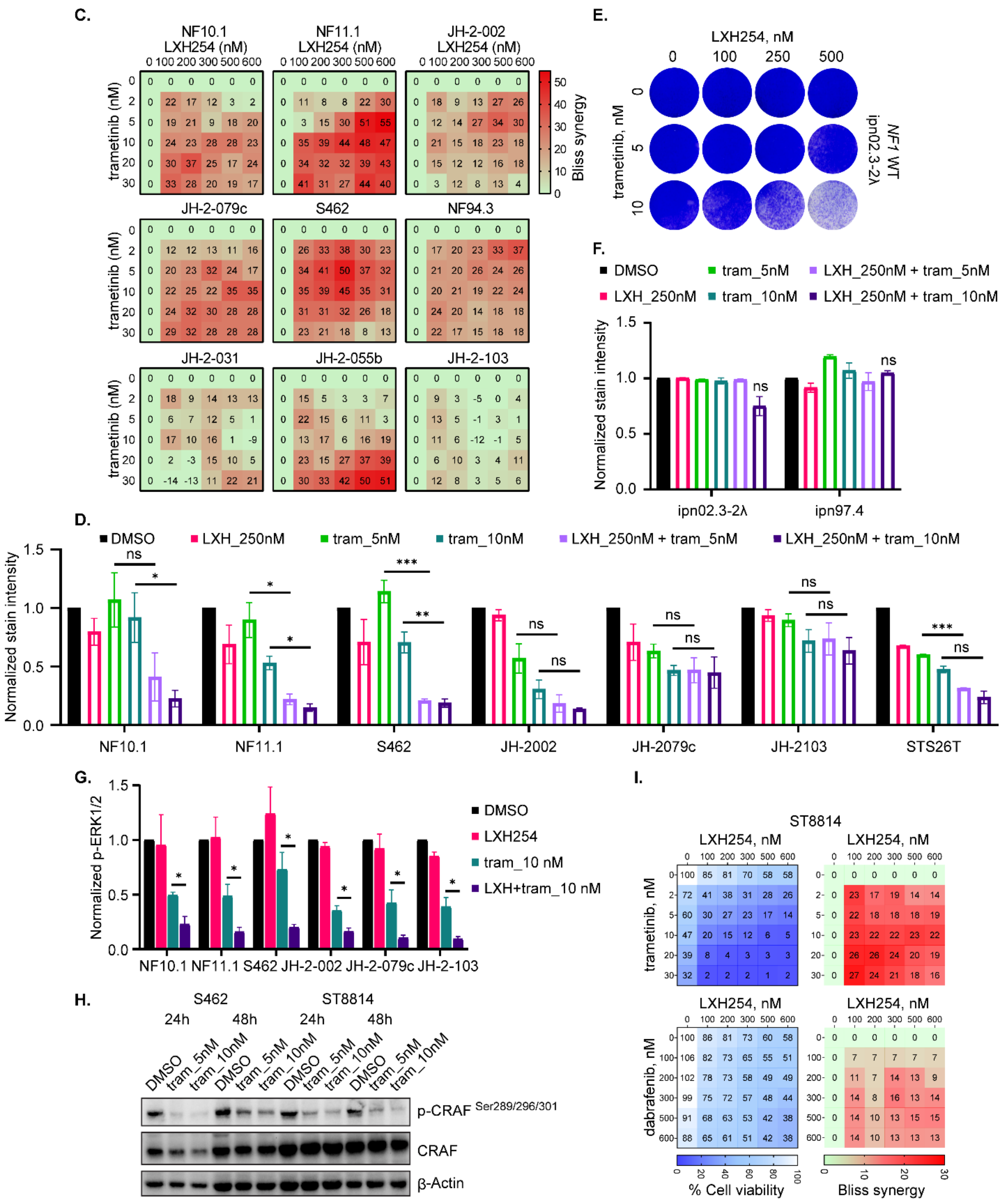
C.
NF10.1 LXH254 (nM)
NF11.1 LXH254 (nM)
JH-2-002 LXH254 (nM)
0 100 200 300 500 600
trametinib (nM)
Bliss synergy
JH-2-079c
S462
NF94.3
JH-2-031
JH-2-055b
JH-2-103
D.
DMSO
LXH_250nM
tram_5nM
tram_10nM
LXH_250nM + tram_5nM
LXH_250nM + tram_10nM
Normalized stain intensity
NF10.1
NF11.1
S462
JH-2002
JH-2079c
JH-2103
STS26T
E.
LXH254, nM
0 100 250 500
trametinib, nM
0 5 10
NF1 WT
ipn02.3-2λ
F.
DMSO
tram_5nM
LXH_250nM + tram_5nM
LXH_250nM
tram_10nM
LXH_250nM + tram_10nM
Normalized stain intensity
ipn02.3-2λ
ipn97.4
G.
Normalized p-ERK1/2
DMSO
LXH254
tram_10 nM
LXH+tram_10 nM
NF10.1 NF11.1 S462 JH-2-002 JH-2-079c JH-2-103
H.
S462
ST8814
24h 48h 24h 48h
DMSO tram_5nM tram_10nM
p-CRAF Ser289/296/301
CRAF
β-Actin
I.
ST8814
LXH254, nM
trametinib, nM
dabrafenib, nM
% Cell viability
Bliss synergy

Figure S4

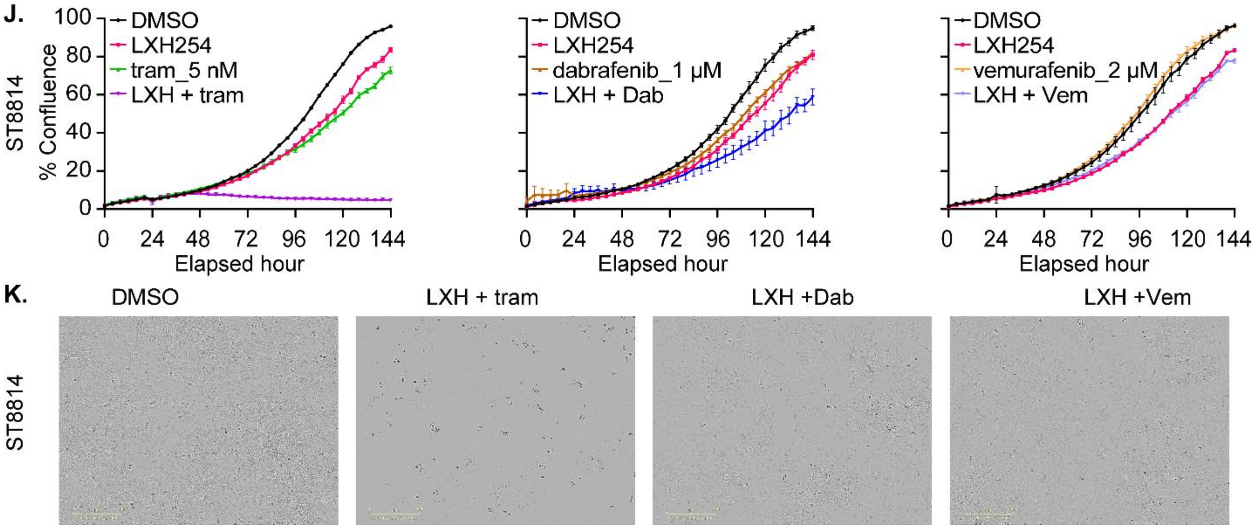

Figure S4. **A**. Bar graphs summarizing percent confluency derived from IncuCyte phase contrast imaging of NF1-MPNST cells treated with LXH254 and/or trametinib as in Figure 4A. Graphs correspond to Figure 4A, and percent confluency at the endpoint (160h) was normalized to the confluency of DMSO treated cells. Error bars represent SEM from three biological replicates with three technical replicates in each setup. Trametinib treated groups were compared to combination treated groups and statistical difference was determined using unpaired Student t-test with GraphPad Prism software. **** p<0.0001. **B**. Representative images from IncuCyte phase contrast imaging of NF1-MPNST cells treated with LXH254 and/or trametinib. Images correspond to the data presented in Figures 4A and S4A. **C**. % cell viability obtained from Figure 4B was analyzed and calculated for bliss synergy scores using the Combenefit software. **D**. Bar graphs summarizing quantification of crystal violet stain intensity of MPNST cell lines treated with LXH254, trametinib, or combinations. Graphs correspond to Figure 4C. Error bars represent SEM from two to three biological replicates. Trametinib-treated groups were compared to combination-treated groups and statistical difference was determined using unpaired t-test with GraphPad Prism software. *p< 0.05, ** p<0.01, *** p<0.001. **E**. *NF1* WT ipn02.3-2λ Schwann cell line was treated and analyzed as described in Figure 4C. Shown are representative images from two biological replicates. **F**. Bar graphs summarizing quantification of crystal violet stain intensity of *NF1* WT Schwann cell lines shown in Figures 4C and S4E. Error bars represent SEM from two biological replicates. Drug-treated groups were compared to DMSO-treated groups and statistical difference was determined using unpaired t-test with GraphPad Prism, ns= not significant. **G.** Bar graphs summarizing p-ERK protein levels normalized to GAPDH levels in NF1-MPNST cells treated with LXH254, trametinib or their combination. Graphs correspond to Figure 4D. Protein levels were quantified from blots using ImageJ. Error bars represent SEM from two biological replicates. In each cell line, trametinib-treated group was compared to combination-treated group and statistical difference was determined using unpaired t-test with GraphPad Prism software. * p<0.05. **H**. Two NF1-MPNST cell lines S462 and ST8814 were treated with DMSO, 5 and 10 nM trametinib for 24 and 48 hours, respectively. The phosphorylation sites associated with CRAF inactivation (p-CRAF Serine 289/296/301), total CRAF and the internal loading control ß-Actin were detected using immunoblot. **I.** ST8814 cells were treated with DMSO, increasing doses of LXH254, trametinib or dabrafenib for 5 days. Cell viability was determined

using the MTT assay and bliss synergy scores were calculated using the Combenefit software. Shown is representative data from two biological replicates with three technical replicates in each setup. **J** and **K**. ST8814 cells were exposed to DMSO, 250 nM LXH254, 5 nM trametinib, 1 µM dabrafenib, 2 µM vemurafenib or their combination as shown for 144 hours. Cell confluence was monitored using IncuCyte (J) and representative cell images reflecting confluence at 3 days of treatment were shown (K). Shown is representative data from two biological replicates with three technical replicates in each setup.

Figure S5

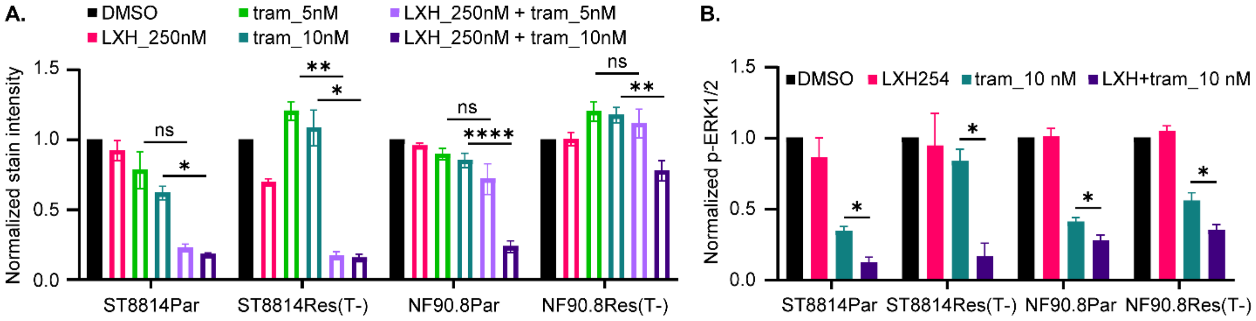

Figure S5. A. Bar graphs summarizing quantification of crystal violet stain intensity of parental and trametinib resistant cell lines treated with LXH254, trametinib or their combinations. Graphs correspond to Figure 5C. Error bars represent SEM from two biological replicates. Trametinib-treated groups were compared to combination-treated groups and statistical difference was determined using unpaired t-test with GraphPad Prism software. * $p< 0.05$, ** $p< 0.01$, **** $p<0.0001$, ns= not significant. **B**. Bar graphs summarizing p-ERK protein levels normalized to GAPDH levels in parental and trametinib resistant cell lines treated with LXH254, trametinib or their combination. Graphs correspond to Figure 5D. Protein levels were quantified from blots using ImageJ. Error bars represent SEM from two biological replicates. In each cell line, trametinib-treated group was compared to combination-treated group and statistical difference was determined using unpaired t-test with GraphPad Prism software. * $p<0.05$.

Figure S6

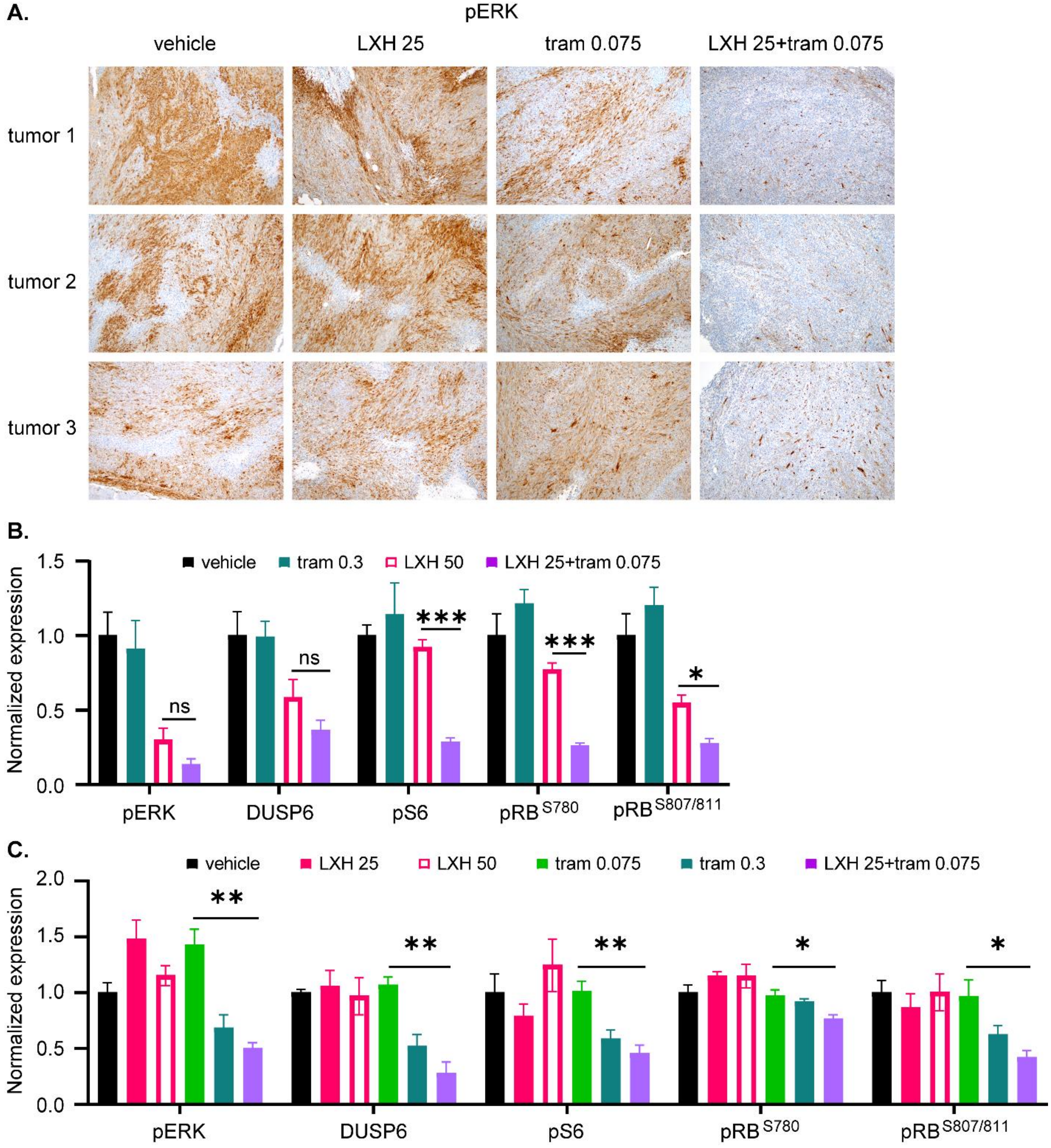

Figure S6. **A**. Additional images of pERK IHC staining as shown in Figure 6C. Images of all pERK staining performed across 4 conditions, 3 tumors/ condition (4x3 grid) are shown. B-C. Bar graphs summarizing pERK, DUSP6, pS6, pRB protein levels normalized to actin levels in MA1334 (B) and GAPDH levels in JH-2-002 (C) xenograft models. Graphs correspond to Figures 6E and 6F respectively. Protein levels were quantified from blots using ImageJ. Error bars represent SEM from the three biological replicates shown in the blots. In each xenograft, single agent-treated group was compared to combination-treated group and

statistical difference was determined using unpaired t-test with GraphPad Prism software. * $p<0.05$, ** $p<0.01$, *** $p<0.001$, ns = not significant.

Supplementary Table S1. Cell line, antibody and reagent list

See the excel file named "Table S1_Cell line Antibody and Reagent list" for specific details.

Supplementary Table S2. Quality control metrics for RNAseq

See the excel file named "Table S2_Quality control metrics for RNAseq" for details related to Figure 5E.

Supplementary Table S3. Mouse tumor volumes

See the excel file named "Table S3_Mouse tumor volumes" for the source data related to Figure 6.

Supplementary References

1. Wang J, Calizo A, Zhang L, Pino JC, Lyu Y, Pollard K, et al. CDK4/6 inhibition enhances SHP2 inhibitor efficacy and is dependent upon RB function in malignant peripheral nerve sheath tumors. Sci Adv. 2023;9(47):eadg8876.
2. Pollard K, Banerjee J, Doan X, Wang J, Guo X, Allaway R, et al. A clinically and genomically annotated nerve sheath tumor biospecimen repository. Sci Data. 2020;7(1):184.
3. Wang J, Pollard K, Calizo A, Pratilas CA. Activation of Receptor Tyrosine Kinases Mediates Acquired Resistance to MEK Inhibition in Malignant Peripheral Nerve Sheath Tumors. Cancer Res. 2021;81(3):747-62.
4. Monaco KA, Delach S, Yuan J, Mishina Y, Fordjour P, Labrot E, et al. LXH254, a Potent and Selective ARAF-Sparing Inhibitor of BRAF and CRAF for the Treatment of MAPK-Driven Tumors. Clin Cancer Res. 2021;27(7):2061-73.
5. Rabara D, Tran TH, Dharmaiah S, Stephens RM, McCormick F, Simanshu DK, et al. KRAS G13D sensitivity to neurofibromin-mediated GTP hydrolysis. Proc Natl Acad Sci U S A. 2019;116(44):22122-31.
6. Garcia N, Del Pozo V, Yohe ME, Goodwin CM, Shackleford TJ, Wang L, et al. Vertical Inhibition of the RAF-MEK-ERK Cascade Induces Myogenic Differentiation, Apoptosis, and Tumor Regression in H/NRAS(Q61X) Mutant Rhabdomyosarcoma. Mol Cancer Ther. 2022;21(1):170-83.
7. He F, Bandyopadhyay AM, Klesse LJ, Rogojina A, Chun SH, Butler E, et al. Genomic profiling of subcutaneous patient-derived xenografts reveals immune constraints on tumor evolution in childhood solid cancer. Nat Commun. 2023;14(1):7600.
8. Krueger F, James F, Ewels P, Afyounian E, Schuster-Boeckler BJZd. FelixKrueger/TrimGalore: v0. 6.7-doi via zenodo. 2021;10.
9. Harrow J, Frankish A, Gonzalez JM, Tapanari E, Diekhans M, Kokocinski F, et al. GENCODE: the reference human genome annotation for The ENCODE Project. Genome Res. 2012;22(9):1760-74.
10. Ahdesmaki MJ, Gray SR, Johnson JH, Lai Z. Disambiguate: An open-source application for disambiguating two species in next generation sequencing data from grafted samples. F1000Res. 2016;5:2741.
11. Li B, Dewey CN. RSEM: accurate transcript quantification from RNA-Seq data with or without a reference genome. BMC Bioinformatics. 2011;12:323.
12. Liberzon A, Subramanian A, Pinchback R, Thorvaldsdottir H, Tamayo P, Mesirov JP. Molecular signatures database (MSigDB) 3.0. Bioinformatics. 2011;27(12):1739-40.
13. Fang Z, Liu X, Peltz G. GSEApy: a comprehensive package for performing gene set enrichment analysis in Python. Bioinformatics. 2023;39(1).
14. Hunter JDJCis, engineering. Matplotlib: A 2D graphics environment. 2007;9(03):90-5.
15. Waskom MLJJoOSS. Seaborn: statistical data visualization. 2021;6(60):3021.
